# GPAHex-A synthetic biology platform for Type IV–V glycopeptide antibiotic production and discovery

**DOI:** 10.1038/s41467-020-19138-5

**Published:** 2020-10-16

**Authors:** Min Xu, Wenliang Wang, Nicholas Waglechner, Elizabeth J. Culp, Allison K. Guitor, Gerard D. Wright

**Affiliations:** David Braley Center for Antibiotic Discovery, M.G. DeGroote Institute for Infectious Disease Research, Department of Biochemistry and Biomedical Sciences, McMaster University, Hamilton, ON Canada

**Keywords:** Antibiotics, Metabolic engineering, Natural product synthesis, Antibiotics

## Abstract

Glycopeptide antibiotics (GPAs) are essential for the treatment of severe infectious diseases caused by Gram-positive bacteria. The emergence and spread of GPA resistance have propelled the search for more effective GPAs. Given their structural complexity, genetic intractability, and low titer, expansion of GPA chemical diversity using synthetic or medicinal chemistry remains challenging. Here we describe a synthetic biology platform, GPAHex (GPA Heterologous expression), which exploits the genes required for the specialized GPA building blocks, regulation, antibiotic transport, and resistance for the heterologous production of GPAs. Application of the GPAHex platform results in: (1) a 19-fold increase of corbomycin titer compared to the parental strain, (2) the discovery of a teicoplanin-class GPA from an *Amycolatopsis* isolate, and (3) the overproduction and characterization of a cryptic nonapeptide GPA. GPAHex provides a platform for GPA production and mining of uncharacterized GPAs and provides a blueprint for chassis design for other natural product classes.

## Abstract (web summary)

Expansion of the chemical diversity of glycopeptide antibiotics (GPAs) to deal with the emergence and spread of GPA resistance is challenging. Here, the authors report a GPA synthetic biology platform in *Streptomyces coelicolor* for Type IV–V glycopeptide antibiotic production and discovery.

## Introduction

**Fig. 1:**
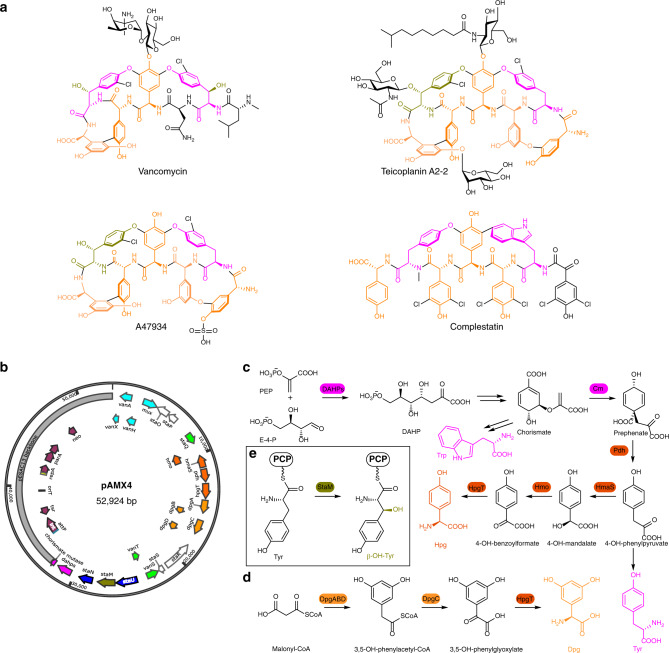
GPA representatives and biosynthesis of aromatic building blocks for GPAs. **a** Representatives of GPAs. **b** pAMX4 bearing the GPA synthetic cassettes originated from the A47934 BGC for making the GPAHex production chassis. Genes are colored according to their functions: cyan (resistance), white (unknown), green (regulation), dark orange (Hpg biosynthesis), orange (Dpg biosynthesis), blue (transportation), olive (Bht biosynthesis), pink (Tyr/Trp/Hpg biosynthesis), and gray (pESAC13 plasmid backbone). **c** Biosynthetic pathway for producing Trp, Tyr, and Hpg. **d** Biosynthetic pathway for producing Dpg. **e** Biosynthetic pathway on pAMX4 for producing Bht. Aromatic amino acids are colored according to their biosynthetic genes.

Glycopeptide antibiotics (GPAs), such as vancomycin and teicoplanin (Fig. 1a), are microbial natural products (NPs) that are essential for the control of infections caused by Gram-positive pathogens. Vancomycin and teicoplanin inhibit bacterial cell wall biosynthesis by binding to the d-Alanine-d-Alanine terminating dipeptide of growing peptidoglycan and its extracellular precursors. Like all antibiotics, GPAs are vulnerable to resistance^1^. Vancomycin resistance occurs through reprogramming of cell wall biosynthesis, mediated by a *vanRSHAX* resistance cassette that replaces the d-Ala-d-Ala termini with d-Ala-d-Lactate. This change of an amide linkage to an ester decreases the affinity of vancomycin to the d-Ala-d-Ala terminus by 1000 fold, resulting in a commensurate increase in the antibiotic minimal inhibitory concentration (MIC)^2–4^. This mechanism dominates in resistant enterococci, while in *Staphylococcus aureus*, thickening of the cell wall, presumably accompanied by an increase in d-Ala-d-Ala termini that can adsorb increased levels of antibiotic, results in elevated tolerance to vancomycin^5^.

The spread of vancomycin resistance across the globe and in most health care settings warrants the investigation of GPAs with improved efficacy. To this end, three semi-synthetic derivatives of NP GPAs, telavancin, dalbavancin, and oritavancin, have been successfully introduced to the clinic^6–11^. Despite this success, access to next-generation GPAs remains challenging. On reason for this difficulty is the chemical complexity of GPAs, which remains difficult to approach by total synthesis on a scale sufficient for clinical trials. Furthermore, NP GPAs are often difficult to acquire due to poor fermentation titers. These challenges restrict the opportunities of securing GPAs for exploration as drug candidates^12,13^.

While most GPAs target the d-Ala-d-Ala terminus of peptidoglycan, we recently established that a subgroup, termed Type V GPAs^12^, binds to the cell wall in a distinct manner^14^. Type V GPAs are characterized by a tryptophan (Trp) linked to the central 4-hydroxyphenylglycine (Hpg) and are not generally glycosylated. Because they do not bind d-Ala-d-Ala, they are not susceptible to the canonical d-Ala-d-Lac-mediated GPA resistance and offer a class of promising antibiotics.

GPA core peptide scaffolds are synthesized through large multi-subunit non-ribosomal peptide synthetases (NRPSs)^15,16^. These are arrayed along the genomes of actinobacteria in biosynthetic gene clusters (BGCs) that include genes required for the chemical modification of the peptide, the production of constituent elements, such as precursor amino acids, acyl chains, and sugars, in addition to genes for regulation, transport, and resistance^17^. The adenylation (A) domains of the NRPS modules recognize the amino acid components, many of which are nonproteinogenic. These include Hpg, 3,5-dihydroxyphenylglycine (Dpg), β-hydroxytyrosine (Bht), in addition to tyrosine (Tyr), Trp, leucine, and others. The amino acids are loaded onto the peptidyl carrier protein (PCP) domain of the NRPS for elongation by the condensation domain that catalyzes amide bond formation. During the process of loading, aryl group halogenation and β-hydroxylation on Tyr may also occur, introducing chlorinated amino acids and Bht into GPAs^18,19^. Bht can also be provided by a mini-NRPS pathway comprised of BpsD, OxyD, and Bhp^20^. Upon reaching the last module of the NRPS assembly line, the full-length peptidyl chain tethered to the last PCP domain can be crosslinked through intramolecular biaryl and diphenyl ether bonds. These are formed by cluster-associated cytochrome P450s, each recruited by adjacent characteristic X domain present in the last NRPS module^21^. Crosslinks in the GPA scaffolds confer on these antibiotics a cup-like 3D conformation that can tightly bind to peptidoglycan and its precursors^12^. Following peptide release from the NRPS, additional modifications such as glycosylation, acylation, and sulfation can occur, contributing to GPA chemical diversity^17^.

As a consequence of next-generation genome sequencing technologies, BGCs that may encode GPA scaffolds can be easily identified in actinomycete genomes. The associated BGCs represent an untapped resource for GPA discovery and development^22^. Many of these BGCs are ‘cryptic’, i.e., not expressed under laboratory conditions, or yield only small amounts of compound upon fermentation. Consequently, accessing GPAs remains challenging. Synthetic biology strategies such as heterologous expression in an accommodating chassis offer potential solutions to these difficulties^13^. There are two reports of heterologous expression of GPAs, complestatin and A47934, where the yield of complestatin was only 0.24 mg/L in *Streptomyces lividans* TK24^23,24^. An additional obstacle is the large size of GPA BGCs (often >70 kb), which makes them difficult to clone^15,25^, and the limited options for surrogate chassis strains.

Here, we describe the GPAHex platform based on an engineered GPA production chassis, *S. coelicolor* M1154/pAMX4, and an optimized transformation associated recombination (TAR) system for the production of known GPAs and mining of cryptic GPAs. GPAHex offers a synthetic biology platform for titer improvement and, importantly, to access to GPA chemical ‘dark matter’^26^ embedded in microbial genomes.

## Results

### Construction of the GPAHex platform

We chose to use *S. coelicolor* as the chassis for the GPAHex platform as it is a well-documented surrogate host for actinomycete gene expression, and we had previously shown that it supports good production of the GPA A47934 (~100 mg/L)^24^. Using the P1-phage artificial chromosome (PAC) plasmid pA47934^24^, which contains the A47934 BGC (~67 kbp) and ~70 kbp of unrelated DNA, we removed the NRPS scaffold, the tailoring genes, and the unrelated region using λ-red mediated PCR-targeting^27^. We next mobilized the remaining plasmid into *S. coelicolor* to establish the GPAHex platform (Fig. 1b and Supplementary Fig. 1). Hpg, Tyr, and Trp are all synthesized via the shikimate pathway^17^ (Fig. 1c), which is highly regulated by its intermediates. We, therefore, introduced genes encoding the gatekeepers of the shikimate pathway, 3-deoxy-D-arabino-heptulosonate 7-phosphate (DAHP) synthase and chorismate mutase, from *S. toyocaensis* NRRL15009, downstream from the remaining A47934 biosynthetic genes resulting in plasmid pAMX4 (Fig. 1b and Supplementary Fig. 1). pAMX4 was delivered into *S. coelicolor* M1154^28^ and integrated into the *attB*_*φC31*_ site to generate the GPAHex chassis, *S. coelicolor* M1154/pAMX4.

### Refactoring of the vector for TAR cloning of large NP BGCs

GPAs are synthesized from large 50 kb–90–kb BGCs, which are challenging to manipulate using standard DNA cloning methods. Two examples of GPA BGC cloning: (1) PAC library based random cloning of the A47934 BGC^24^ and (2) λ-red mediated stitching of two cosmids to reassemble the complestatin BGC^23^ have been reported. Both methods required labor-intensive and time-consuming genomic library construction and screening. Moore’s group has pioneered the TAR system for targeted capture of NP BGCs using the in vivo homologous recombination system in yeast^29,30^. However, this method suffers from low capture efficiency and instability in maintaining large and repetitive BGCs such as those of GPAs^31^.

We developed a protocol for cloning of large NP BGCs by constructing vector pCGW, which replaces the SuperCos I backbone of pCAP03-aac(3)IV with the copy number control replicon bearing ‘*oriV-ori2-repE-sopABC*’ cassette from pBAC-lacZ. pCGW can be maintained at 1–2 copies/cell when supplemented with 0.2 % d-glucose; however, it can be conditionally induced to ~100 copies/cell by adding 1 mM l-arabinose in a *trfA*^+^ *E. coil* strains such as *E. coli* EPI300, which are critical for stable maintenance of large exogenous DNA^32^. Significant differences in the concentration of pCGW with and without l-arabinose induction are readily apparent (Supplementary Fig. 2a, b).

Since pCGW was derived from pCAP03-aac(3)IV, we retained the use of the *ura3* counter-selection marker by the insertion of capturing ‘hook sequences’, which are DNA sequences homologous to the two ends of target DNA region, between its TATA box and the ATG start codon^30^. Despite this counter-selection system, we observed high levels of background colonies due to insufficient expression of *ura3*, likely caused by the absence of transcriptional initiation sites (TIS) between the TATA box and start codon. Therefore, when designing the hooks, we included additional TIS sequences (CAAG, CAAA, TAAA, TAAT, or TAAG) to ensure transcription initiation of *ura3*^33^. The *Schizosaccharomyces pombe* pADH promoter has a transcription initiation window of 55–125 bps (75–115 bps is optimal) downstream of the TATA box^33^, so scanning of target BGCs to include the TIS sequences is critical for designing capture hooks. Since transcription of the pADH promoter can be initiated at different TISs, multiple TISs are preferred to maximize *ura3* transcription to reduce background colonies generated by non-homologous end joining, consequently improving the capture efficiency.

### GPAHex production of corbomycin

**Fig. 2:**
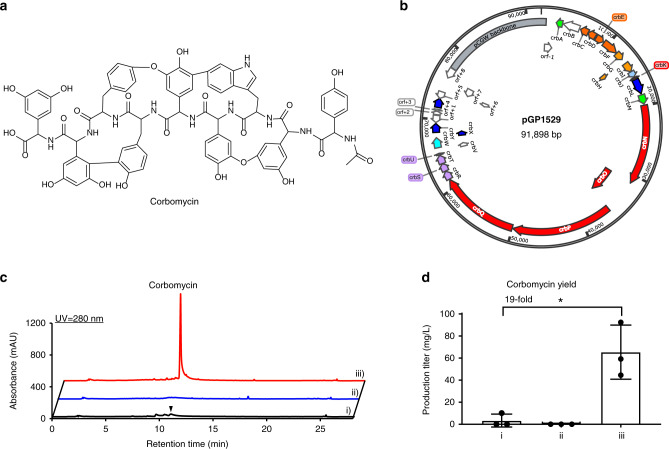
Production of corbomycin using GPAHex. **a** Chemical structure of corbomycin. **b** pGP1529 bearing *crb* BGC captured through TAR. Genes are color-coded as in Fig. 1 with the addition of red (NRPS scaffold and *mbtH* genes) and purple (P450s genes). **c** Heterologous expression of corbomycin using GPAHex. The inverted triangle represents the small corbomycin peak produced by the parental strain *S*. sp. WAC01529 (i)). No corbomycin was detected in *S. coelicolor* M1154 (ii)), while a large corbomycin peak was evident in the GPAHex production chassis’ trace (iii)). **d** Quantification of corbomycin production. Production of corbomycin in the GPAHex production chassis (iii)) was increased 19-fold compared to the parental strain *S*. sp. WAC01529 (ii)). Mean with error bar showing s.d. of three biological replicates (*n* = 3) is plotted. Significance was tested to *P = 0.0131 by an unpaired two-sided Student’s *t*-test. Similar results (**d**) were obtained from two independent experiments.

Corbomycin (Fig. 2a) is a Type V nonapeptide GPA discovered through phylogeny-guided genome mining^14^. While corbomycin shows clinical promise, it has a relatively low yield in the producing strain *Streptomyces* sp. WAC01529 (<4 mg/L) limiting its further development. Given the genetic intractability of *S*. sp. WAC01529, heterologous expression offers an alternative approach to improve the titer and provide a platform for synthetic biology development of corbomycin. The corbomycin peptide scaffold includes three Hpg (Hpg1, Hpg4, and Hpg5), three Dpg (Dpg2, Dpg8, and Dpg9), two Tyr (Tyr6 and Tyr7), and one Trp3 residues. These shikimate pathway or type III polyketide synthase (PKS)^17^ derived aromatic amino acids provide an ideal pressure test for our GPAHex platform.

Genome sequencing of *S*. sp. WAC01529 revealed a ~70 kb *crb* BGC covering 30 genes (Supplementary Table 2). We searched the boundary regions of *crb* BGC and designed a pair of capture hooks bearing five TISs (six TISs in total, including one introduced by the *Pme* I site) (Supplementary Table 1) for cloning a region of 76 kb from the chromosome of *S*. sp. WAC01529 into pCGW. Seven out of ten yeast colonies showed a positive signal by PCR screening—a 70% capture rate (Supplementary Fig. 3a). Plasmid bearing the *crb* BGC, pGP1529 (Fig. 2b), was mobilized into *S. coelicolor* M1154 and *S. coelicolor* M1154/pAMX4 for corbomycin production through *E. coli*-*Streptomyces* intergenic tri-parental mating^34^. Antibacterial activity assay against *Bacillus subtilis* 168 revealed robust growth inhibition from *S. coelicolor* M1154/pAMX4/pGP1529 extracts; no growth inhibition was observed from *S. coelicolor* M1154/pGP1529 extracts (Supplementary Fig. 4). The yields of corbomycin in *S*. sp. WAC01529, *S. coelicolor* M1154/pGP1529, and *S. coelicolor* M1154/pAMX4/pGP1529, determined by high-performance liquid chromatography coupled with quadrupole time-of-flight mass spectrometry (HPLC-QTOF-MS), were 3.4 mg/L, 0 mg/L, and 65.4 mg/L, respectively, indicating a 19-fold increase in titer using the GPAHex platform (Fig. 2c, d).

As noted above, pAMX4 was inserted into the *attB*_*φC31*_ site located at position *SCO3798* on the chromosome of *S. coelicolor* M1154. The pCGW plasmids include the same integration system and are predicted to integrate into the chromosome at pseudo-*attB*_*φC31*_ sites through site-specific recombination or at the pAMX4 site through homologous recombination. There are three pseudo-*attB*_*φC31*_ sites present in *S. coelicolor* located in *SCO3398*, *SCO3792-SCO3793* intergenic region, and *SCO4645*^35^. Using whole-genome sequencing and PCR, pGP1529 was found to be integrated into *S. coelicolor* M1154/pAMX4 at the *attL-int* site downstream of *SCO3797* through homologous recombination between the 2,064 bp *attP-int* region from the two plasmid backbones (Supplementary Fig. 5a, b). Advantageously, homologous recombination integration of pGP1529 positions the *crb* BGC adjacent to the GPA biosynthetic cassettes of pAMX4. The presence of *neo* and *tsr* genes upstream and downstream of the *attL-int* homologous recombination site provides stable maintenance of the GPA BGC with selection by kanamycin and thiostrepton.

### Discovery of GP1416 from *Amycolatopsis* sp. WAC01416

*Amycolatopsis* strains are more common GPA producers in comparison with other actinomycetes^12^; however, there have been no reports of heterologous expression of an *Amycolatopsis* GPA BGC in a *Streptomyces* surrogate host. We previously isolated a series of soil actinomycetes that show resistance to vancomycin, providing an enriched pool of putative GPA producers^36^. Genome sequencing of these isolates revealed dozens of GPA BGCs. Among these strains, *Amycolatopsis* sp. WAC01416 harbors a ~67 kb teicoplanin-like GPA. A domain analysis^37^ predicted a heptapeptide scaffold of ‘Hpg-Bht(or Tyr)-Dpg-Hpg-Hpg-Bht-Dpg’ similar to that of ‘Hpg-Tyr-Dpg-Hpg-Hpg-Bht-Dpg’ heptapeptide of teicoplanin, a type IV lipoglycopeptide. Based on the sequence information (Supplementary Table 3), we predicted a halogenated teicoplanin-type GPA with ‘A-B’, ‘C-O-D’, ‘D-O-E’, and ‘F-O-G’ crosslinks, *N*-acylated-glucosamine on Hpg4, *N*-acetylglucosamine on Bht6, and α-d-mannose on Dpg7 for GP1416. However, we were unable to isolate GPAs from *Amycolatopsis* sp. WAC01416, and so turned to our GPAHex platform to capture and express the GP1416 BGC.

**Fig. 3:**
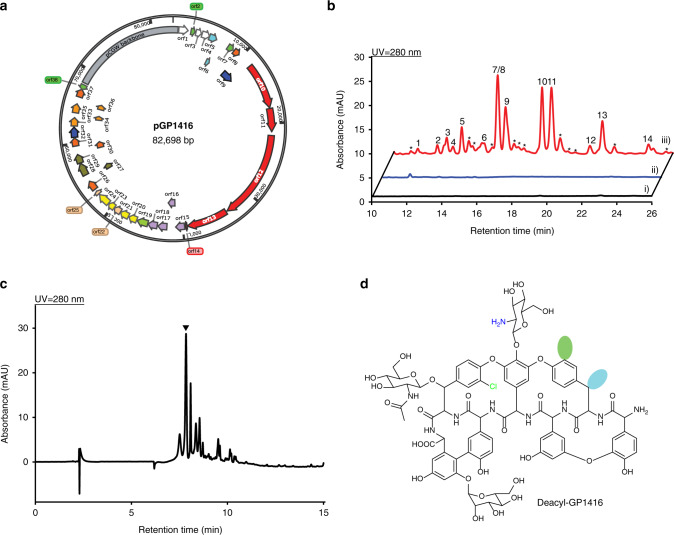
Discovery of the cryptic GP1416 from *Amycolatopsis* sp. WAC01416 using GPAHex. **a** pGP1416 bearing GP1416 BGC captured through TAR. **b** Heterologous expression of GP1416 using GPAHex. GP1416 analogs can only be detected in the GPAHex host (iii)) but not in the parental strain *Amycolatopsis* sp. WAC01416 (i)) or in *S. coelicolor* M1154/pGP1416 (ii)). Peaks 1–14 are labeled and their associated MS spectra and proposed structures are presented in Supplementary Fig. 12. Peaks labeled with asterisks are also GP1416 analogs showing characteristic UV absorption of GPA. **c** Heterologous expression of deacyl-GP1416 using GPAHex. Acyltransferase deletion mutant *S. coelicolor* M1154/pAMX4/pGP1416Δ*orf22* shows less complex GPA production (compare to trace iii) in (**b**). Deacyl-GP1416 labeled with inverted triangle is purified and characterized as shown in (**d**). **d** Chemical structure of deacyl-GP1416. Peptide scaffold of GP1416 varies by the presence of a Tyr, Bht, m-Cl-Tyr, or m-Cl-Bht residue at position AA2 and a Bht or m-Cl-Bht residue at position AA6. Putative chlorination (green) and hydroxylation (cyan) on AA2 are highlighted.

Two capture hooks bearing four TISs were designed to clone GP1416 BGC into pCGW (Supplementary Table 1). Three out of twelve screened yeast colonies showed a positive signal in PCR screening—a 25% capture rate (Supplementary Fig. 3b). Verified plasmid, pGP1416 (Fig. 3a), was mobilized into *S. coelicolor* M1154 and *S. coelicolor* M1154/pAMX4 for GP1416 production through intergenic tri-parental mating. Antibacterial activity assay against *B. subtilis* 168 revealed no growth inhibition from extracts of *Amycolatopsis* sp. WAC01416 or *S. coelicolor* M1154/pGP1416; however, *S. coelicolor* M1154/pAMX4/pGP1416 extracts showed excellent activity (Supplementary Fig. 6). pGP1416 integrates into the same site as pGP1529 on the *S. coelicolor* M1154/pAMX4 chromosome (Supplementary Fig. 7). HPLC analysis of d-Ala-d-Ala affinity column partially purified extracts revealed the presence of a series of peaks in *S. coelicolor* M1154/pAMX4/pGP1416 chromatograms compared to the controls *Amycolatopsis* sp. WAC01416 and *S. coelicolor* M1154/pGP1416 (Fig. 3b). HRESI-QTOF-MS analysis detected a series of mass signals in the partially purified extract, which are distinct from those of teicoplanin (Supplementary Fig. 17). Given the number of closely related analogs produced by *S. coelicolor* M1154/pAMX4/pGP1416, it proved difficult to purify a specific single compound for NMR analysis. Most of the analogs of type IV lipoGPAs are generated by loading various acyl side chains on the N2′ position of the glucosamine attached to Hpg4. We, therefore, deleted the acyltransferase coding gene (*orf22*) on pGP1416 to simplify the metabolic profile and facilitate the purification of deacyl-GP1416 for structural determination (Fig. 3c, Supplementary Fig. 8). HRESI-QTOF-MS revealed an *m/z* value of 1690.4673 ([M + H]^+^) for deacyl-GP1416, indicating a molecular formula of C_78_H_80_ClN_9_O_32_ (Supplementary Fig. 9). The chemical structure of deacyl-GP1416 was characterized as shown in Fig. 3d using further 1D and 2D NMR analyses (Supplementary Fig. 10–15 and Supplementary Table 5). Dechlorinated, dichlorinated, and hydroxylated deacyl-GP1416 analogs were also observed in the mass spectra (Supplementary Fig. 16). Based on the structure of deacyl-GP1416 and the mass spectra of GP1416, predicted structures of GP1416 are shown in Supplementary Fig. 17. The key differences between GP1416 and teicoplanin are in the chlorination of Tyr2 and Bht6, hydroxylation on Tyr2, and the acyl chain on glucosamine attached to Hpg4, which contribute to the complex metabolic profile of GP1416.

No GP1416 compounds were detected in *Amycolatopsis* sp. WAC01416, suggesting that the GP1416 BGC is a cryptic cluster. This hypothesis was confirmed using reverse transcription-polymerase chain reaction (RT-PCR) analysis. The GP1416 BGC was transcriptionally inactive in *Amycolatopsis* sp. WAC01416 and *S. coelicolor* M1154/pGP1416 but actively transcribed in the GPAHex production host, *S. coelicolor* M1154/pAMX4 (Supplementary Fig. 18).

### Discovery of a cryptic Type V GPA-GP6738

In our phylogenetic analysis of GPA biosynthesis and resistance^22^, we discovered a clade of *Streptomyces* strains harboring an almost identical BGC encoding a Type V GPA (Supplementary Fig. 19). A domain analysis^37^ predicted a nonapeptide scaffold of ‘Dpg-Dpg-Val-Trp-Dpg-Hpg-Dpg-Tyr-Dpg’. Analysis of the two P450 enzymes in the BGC revealed that they are closely related to ComI and ComJ in complestatin’s BGC (Supplementary Fig. 20), suggesting two possible crosslinks between Trp4 and Hpg6, and Hpg6 and Tyr8 residues. Unfortunately, when we attempted to isolate the compound from the wild type strains, *Streptomyces* sp. WAC06738, *Streptomyces* sp. CNQ329, *Streptomyces* sp. CNQ509, *Streptomyces* sp. CNQ525, *Streptomyces* sp. CNQ865, *Streptomyces* sp. CNT371, and *Streptomyces* sp. CNY243, no related compounds were detected after testing against a panel of different media, consistent with the ‘cryptic’ designation of GP6738. All of these strains are slow-growing and genetically intractable, making GP6738 a candidate for our GPAHex platform.

**Fig. 4:**
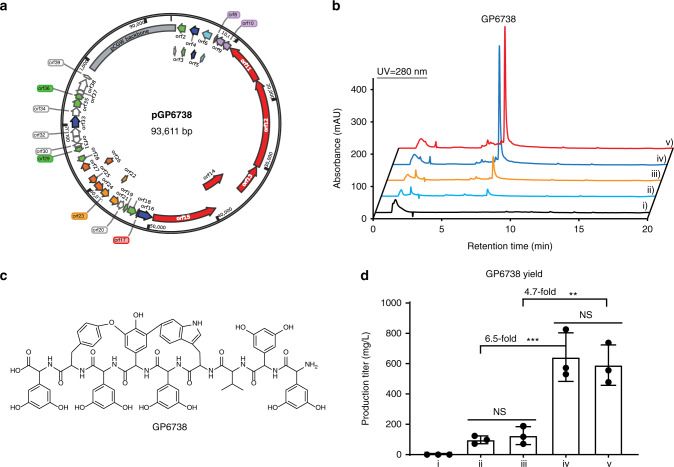
Discovery of the cryptic GP6738 from *S.* sp. WAC06738 using GPAHex. **a** pGP6738 bearing GP6738 BGC captured through TAR. Genes are color-coded as in Figs. 1 and 2. **b** Heterologous expression of GP6738 using GPAHex. No GP6738 was detected in the parent *S*. sp. WAC06738 (i)). A prominent GP6738 peak was detected in all heterologous expression strains: *S. coelicolor* M1154/pGP6738 (ii)), *S. coelicolor* M1154/pAMX4/pGP6738 (iii)), *S. coelicolor* M1154/pGP6738/pIJ10257-sl (iv)), and *S. coelicolor* M1154/pAMX4/pGP6738/pIJ10257-sl (v)). Overexpression of the pathway-situated regulators highly increased GP6738 production. **c** Chemical structure of GP6738. GP6738 bears a rare nonapeptide scaffold harboring a Val3 residue and the characteristic Tyr-Hpg-Trp dual crosslinks of Type V GPAs. **d** Quantitation of GP6738 production. Overexpression of *strR-lmbU* regulators increased the yield of GP6738 by 6.5-fold in *S. coelicolor* M1154 and 4.7-fold in the GPAHex production chassis. Mean with error bars showing s.d. of three biological replicates (*n* = 3) is plotted. Multiple comparison significance was tested to ***P = 0.0003 or **P = 0.0005 by one-way ANOVA with Turkey’s post hoc analysis. NS, not significant. Similar results (**d**) were obtained from two independent experiments.

The GP6738 BGC was cloned through TAR as mentioned above, resulting in plasmid pGP6738 (Fig. 4a), followed by introducing into *S. coelicolor* M1154 and *S. coelicolor* M1154/pAMX4 for expression. pGP6738 integrates into the identical site as pGP1529 and pGP1416 on *S. coelicolor* M1154/pAMX4 chromosome (Supplementary Fig. 21). Antibacterial activity assay against *B. subtilis* 168 revealed no growth inhibition from the *S*. sp. WAC06738 extracts, in contrast, robust growth inhibition was observed from the *S. coelicolor* M1154/pGP6738 and *S. coelicolor* M1154/pAMX4/pGP6738 extracts (Supplementary Fig. 22). HPLC analysis also revealed a distinct peak with characteristic GPA UV absorbance at 280 nm (Fig. 4b). HRESI-QTOF-MS analysis of the differential peak revealed the accurate mass of 1437.4603 ([M + H]^+^), matching the predicted nonapeptide scaffold with two crosslinks (predicted mass of [M + H]^+^ = 1437.4588) (Supplementary Fig. 23a). The good yield of GP6738 by GPAHex enabled purification of 70 mg of GP6738 for structure determination by 1D and 2D NMR (Supplementary Figs. 24–28, Supplementary Table 6). The structure of GP6738 was further confirmed through tandem MS/MS spectrometry (Supplementary Fig. 23b). GP6738 is a type V GPA bearing a ‘Dpg-Dpg-Val-Trp-Dpg-Hpg-Dpg-Tyr-Dpg’ nonapeptide scaffold as predicted, which is modified through a biaryl crosslink between Trp4 and Hpg6, and a diphenyl ether crosslink between Hpg6 and Tyr8 (Fig. 4c). The two intramolecular crosslinks are identical to that of the Type V GPAs complestatin, kistamicin, and corbomycin^12,14^. Unlike all other reported Type V GPAs to date, which are composed solely of aromatic amino acids, GP6738 includes an aliphatic Val3 residue.

Since GP6738 is cryptic in *S*. sp. WAC06738 and there are two predicted positive regulator genes orthologous to *strR* and *lmbU* present in its BGC, we hypothesized that overexpression of these regulators might further increase the expression of the BGC and increase the yield of GP6738^38,39^. We constructed a pIJ10257-derived plasmid bearing *orf10* (*lmbU*) and *orf11* (*strR*) from GP6738’s BGC under the control of the constitutive strong *Streptomyces* promoter ermEp*. This construct was mobilized into *S. coelicolor* M1154/pGP6738 and *S. coelicolor* M1154/pAMX4/pGP6738, and inserted site-specifically at *attB*_*φBT1*_ site. The yields of GP6738 in *S. coelicolor* M1154/pGP6738, *S. coelicolor* M1154/pAMX4/pGP6738, *S. coelicolor* M1154/pGP6738/pIJ10257-sl and *S. coelicolor* M1154/pAMX4/pGP6738/pIJ10257-sl were 98 mg/L, 126 mg/L, 643 mg/L, and 591 mg/L, respectively (Fig. 4b, d). Overexpression of the positive regulators from the GP6738 BGC increased the yield of GP6738 by 6.5- and 4.7-fold in *S. coelicolor* M1154/pGP6738 and *S. coelicolor* M1154/pAMX4/pGP6738. Transcriptional analysis revealed that the GP6738 BGC was transcriptionally inactive in *S*. sp. WAC06738 but actively transcribed in the heterologous expression strains *S. coelicolor* M1154/pGP6738 and *S. coelicolor* M1154/pAMX4/pGP6738 (Supplementary Fig. 29). Overexpression of the pathway-situated regulators *strR* and *lmbU* further increased the expression of the BGC, contributing to a higher yield of GP6738.

### Antibiotic activity of GP6738

**Fig. 5:**
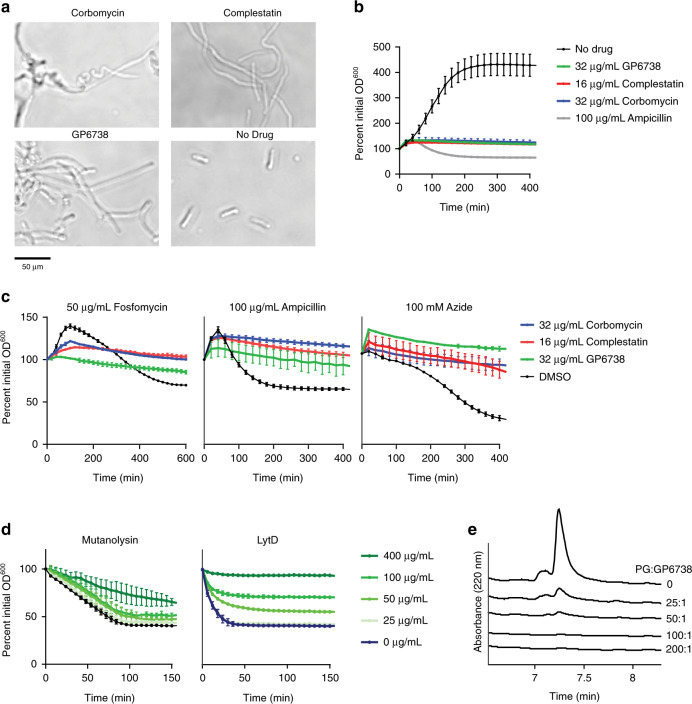
Mode of action study of GP6738. **a** Brightfield microscopy shows the effect of GP6738 on *B. subtilis* 168 morphology. Cells were treated with 0.6× MIC antibiotic (0.6 µg/mL complestatin, 0.6 µg/mL corbomycin, and 3.2 µg/mL GP6738) and grown to mid-log phase. **b** Tracking growth of *B. subtilis* 168 treated with 10× MIC various antibiotics shows their bacteriostatic or bacteriolytic effects. **c** Autolysin inhibitors antagonize lysis of *B. subtilis* 168 treated with peptidoglycan synthesis inhibitors fosfomycin or ampicillin, or the metabolic poison sodium azide. Cells were treated with 10× MIC complestatin, corbomycin, GP6738, or solvent control (DMSO) at the same time that the lytic agent was applied. **d** In vitro digestion of *B. subtilis* peptidoglycan in the presence of GP6738. Insoluble peptidoglycan soaked in various concentrations of GP6738 is then digested with peptidoglycan hydrolases mutanolysin or LytD, and solubilization is tracked by measuring OD_600_. Average values and s.d. from triplicate experiments (*n* = 3) are plotted for panels **b**–**d**. **e** HPLC chromatograms of GP6738 left unbound after incubation with *B. subtilis* peptidoglycan. Antibiotic and peptidoglycan were combined in various w/w ratios, and the decreasing intensity of a peak in comparison to the buffer control that lacked peptidoglycan represents compound binding and removal from solution. Similar results (**a**) were obtained from two independent experiments. Source data underlying Fig. 5a are provided as a Source data file.

Given that GP6738 is a cryptic GPA discovered in this work, we were interested in its bioactivity and mode of action (MOA). MIC determination against a panel of indicator strains revealed a 2–8 and 2–16 -fold higher MIC of GP6738 compared to other Type V GPAs, complestatin, and corbomycin, respectively (Supplementary Table 7). Complestatin and corbomycin inhibit bacteria growth by blocking the action of autolysins, which are essential peptidoglycan hydrolases required for cell growth and division^14,40^. To test whether GP6738 retained the same MOA, we grew *B. sublitis* 168 with sub-MIC of GP6738, resulting in the characteristic elongated cell morphology of autolysin inhibition (Fig. 5a). Further incubation of *B. subtilis* 168 at a concentration of 10-fold MIC of GP6738 showed a bacteriostatic phenotype (Fig. 5b), and GP6738 was able to block cell lysis induced with various agents (fosfomycin, ampicillin, and sodium azide). Like complestatin and corbomycin, GP6738 blocks cell wall digestion by the peptidoglycan hydrolases mutanolysin and LytD in vitro (Fig. 5c–e). GP6738 with complestatin and corbomycin constitute a distinct functional class of GPAs blocking the action of autolysins by binding to peptidoglycan. Additional studies are required to decipher the precise binding site of GP6738 on peptidoglycan.

## Discussion

In contrast to many other classes of antibiotics where multiple ‘generations’ of compounds have been developed over the years to address resistance and improve drug-like qualities, only two NP GPAs: vancomycin and teicoplanin, have dominated clinical use since the discovery of vancomycin in the early 1950s^12^. In recent years, three second-generation semi-synthetic GPAs: telavancin, dalbavancin, and oritavancin, have been introduced that overcome inducible vancomycin resistance and modulate pharmacodynamic parameters of the class^11^. These five compounds represent the complete portfolio of clinically approved GPAs. Reasons for the comparative sparsity of GPA analogs include the chemical complexity of GPAs, which makes total synthesis challenging on a production scale (though creative strategies by the Boger lab have begun to address this limitation)^41^, the rarity of reports of NP GPAs, and the general poor yield of such compounds obtained by fermentation even once they have been discovered.

We developed the GPAHex platform to address many of these drawbacks that plague the discovery and development of GPAs. Using the well-established *S. coelicolor* M1154, we added genes for the supply of specialized biosynthetic precursors (Hpg, Dpg, Bht, Tyr, and Trp), GPA resistance, a cluster-situated positive regulator, and transporters from the A47934 BGC. The chassis *S. coelicolor* M1154/pAMX4 serves as a common stage for the expression of GPA BGCs. A further advancement is the optimization of the TAR cloning method for cloning large specialized metabolite (SM) BGCs using an engineered copy number controlled capture vector pCGW.

Our results show that using the pCGW vector combined with introducing TISs into the cluster capture hooks, the cloning efficiency can reach as high as 70% for large (76 kb) BGCs like *crb*. The introduction of extra TISs helps to increase the transcription of the counter-selection marker gene *ura3* thereby decreasing background colonies from re-circularized plasmid in *Saccharomyces cerevisiae*. The pCGW-based TAR cloning system should also be applicable to other BGCs, accelerating genome mining of cryptic NPs.

The application of GPAHex in the cloning and expression of the Type V GPA corbomycin increased production >19-fold over the parental strain. This recombinant strain provides sufficient material for our ongoing downstream medicinal chemistry program to develop more effective semi-synthetic corbomycin derivatives. Application of GPAHex in the *Amycolatopsis* sp. WAC01416 demonstrated the ability of the platform to express *Amycolatopsis*-derived cryptic BGC in *Streptomyces*. Given the lack of *strR* homolog in *crb* BGC and the silence of the *strR* homolog in GP1416 BGC, the boosted production of corbomycin and activation of GP1416 probably result from the StrR-like regulator StaQ introduced in the GPAHex production chassis as multiple putative StrR binding sites are identified in both BGCs (Supplementary Fig. 30).

In addition to the success of the production of corbomycin and discovery of GP1416, application of GPAHex to *S*. sp. WAC06738 identified a cryptic Type V GPA bearing a nonapeptide scaffold with a unique Val3 residue. Production of GP6738 reaches 125 mg/L in the GPAHex production chassis, which was further improved by >4-fold by overexpression of cluster-situated positive regulators. MOA studies revealed that, like other recently discovered Type V GPAs^14^, GP6738 interrupts the cell wall degradation process by indirectly inhibiting autolysins, which is a promising target for antibiotic drug development.

The GPAHex platform offers a blueprint for further NP research. For example, phenylglycines (Hpg and Dpg) are present not only in GPAs but in a variety of peptide NPs, including nocardicin, feglymycin, enduracidin, ramoplanin, arylomycin and others^42^, GPAHex may be applied for the production and discovery of other NPs. We note that additional synthetic biology tools can be integrated into the GPAHex platform due to the reserved integration sites (ΦBT1, pSAM2, SV1, and R4)^43–46^ in the chromosome for developing novel GPAs.

The GPAHex described here provides a synthetic biology platform for the titer improvement of GPAs and for the discovery of cryptic GPAs in actinomycetes. Success in the discovery and production of GP1416 from *Amycolatopsis* sp. WAC01416 shows that the genetically tractable *Streptomyces* model system is also suitable for the mining of NPs from *Amycolatopsis* strains, which, together with *Streptomyces*, cover ~80% of known GPA BGCs^12,22^. Notably, the GPAHex platform includes most general precursor supply, resistance, transport, and regulation genes for GPA production, however, the offline Bht biosynthetic cassette (*bpsD-oxyD-bhp*) and the amino sugar biosynthetic cassette (*evaABCDE*)^17^, which are necessary for the aglycone scaffold biosynthesis and post-aglycone modification of Type I, II, & III GPAs, were not included in this version of the platform. Further implementation of these gene cassettes into the GPAHex platform in the future would make the platform more general to all GPAs. The modified TAR cloning system provides a significant addition to the synthetic biology toolbox for manipulating large NP BGCs, and the chassis developed here also provides an important tool for targeting of phenylglycine-containing NPs. We predict that this strategy for the development of NP production chassis by targeting genes necessary for precursors production, regulation, resistance, and transport into the chromosome should generally apply to other important NPs such as polyketides and terpenoids obviating the need to work with specific production strains that tend to be limited by genetic tools and inaccessibility.

## Methods

### Strains and plasmids

All strains and plasmids used in this study are listed in Supplementary Data 1.

### Oligonucleotides and reagents

Oligonucleotides used in this study are listed in Supplementary Data 2. gBlocks designed for capturing of GPA BGCs are listed in Supplementary Table 1. Oligonucleotides and gBlocks were ordered from Integrative DNA Technologies (Coralville, IA, USA) and Sanger and genome sequencing were performed at the MOBIX Lab Central Facility (McMaster University). PCR reactions were performed using Dream Taq Green PCR Master Mix (2×) and Phusion Hi-Fidelity DNA polymerase (Thermo Fisher Scientific). Plasmids were purified using the GeneJet Plasmid Miniprep Kit (Thermo Fisher Scientific) except for pGP1529, pGP1416, and pGP6738 which were purified using the alkaline lysis method. Restriction enzymes were purchased from Thermo Fisher Scientific and T4 DNA ligase was purchased from New England Biolabs.

### Growth conditions

*E. coli* was grown at 37 °C, 250 rpm, in LB broth (Bioshop, Canada), and 1 mM of l-arabinose was added to induce the copy number of pCGW derived plasmids in *E. coli* EPI300. Yeast transformants were selected on SD-Trp-5-FOA medium (182 g sorbitol, 20 g glucose, 20 g agar, 880 mL ddH_2_O). After autoclaving, add 10× yeast nitrogen base (100 mL): 1.7 g yeast nitrogen base without amino acids and ammonium sulfate, 5 g ammonium sulfate, 0.832 g amino acid mix without trypophan, 100× adenine (10 mg/mL), and 100× 5-FOA (100 mg/mL)). Positive yeast transformants were cultured in SD-Trp liquid selective medium (182 g sorbitol, 20 g glucose, 890 mL ddH_2_O. After autoclaving, add 10× yeast nitrogen base and 100× adenine). *Streptomyces* strains were grown at 30 °C, 250 rpm, in TSBY medium (30 g tryptone soy broth, 5 g yeast extract, ddH_2_O 1 L) for genomic DNA isolation and seed cultures preparation, and on Soya Flour Medium (SFM, 20 g soya flour, 20 g d-mannitol, 20 g agar, ddH_2_O 1 L, pH 7.2–7.4) for sporulation and conjugation (20 mM MgCl_2_ was added). Fermentation was performed in Streptomyces Antibiotic Activity Medium (SAM, 15 g glucose, 15 g soytone, 5 g NaCl, 1 g yeast extract, 1 g CaCO_3_, 2.5 mL glycerol, ddH_2_O 1 L, pH 6.8). Antibiotics were supplemented as necessary (100 μg/mL ampicillin, 50 μg/mL kanamycin, 50 μg/mL apramycin, 25 μg/mL chloramphenicol, 25 μg/mL thiostrepton, 25 μg/mL nalidixic acid, 50 μg/mL trimethoprim, 50 μg/mL hygromycin for *Streptomyces* and 150 μg/mL hygromycin for *E. coli*).

### Construction of the GPAHex production chassis

The boundary sequence of PAC pA47934^24^ was determined by terminal sequencing using pESAC13-sF primer, including a region of 70,139 bps downstream of the A47934 BGC unrelated to A47934 biosynthesis. This region was deleted using λ-red mediated PCR targeting and a DAHP synthase and choristmate mutase (DAHP-CM) dual gene cassette amplified from *S. toyocaensis* NRRL15009 was introduced in situ. To remove the non-GPA region and introduce DAHP-CM dual cassette, three amplicons were generated. pA4-DAHPs-F/R primers and pA4-CM-F/R primers were used to amplify the DAHP synthase gene and the chorismate mutase gene from *S. toyocaensis* NRRL15009 genome, respectively, and pA4-cat-F/R primers were used to amplify the *cat* gene from pKD3. The three amplicons were stitched together through overlap-extension PCR, resulting in a DAHP-CM-*cat* cassette positioning *Pme*I and *Swa*I sites at the two sides of *cat* gene. The DAHP-CM-*cat* cassette was transformed into *E. coli* BW25113/pKD46/pA47934 strain through electroporation, resulting in pAMX1. Error-free pAMX1 plasmid was digested with *Pme*I and *Swa*I to remove the *cat* gene, and then self-ligated at 20 °C, overnight, using T4 DNA ligase, resulting in pAMX2. The A47934 NRPS and crosslinking genes (*staA-staL*) were deleted using an identical procedure. pA4-Δnrps-F/R primers were used to amplify the *cat* gene from pKD3, followed by transformation into *E. coli* BW25113/pKD46/pAMX2 strain through electroporation, resulting in pAMX3. pAMX3 was digested with *Pme*I and *Swa*I to remove the *cat* gene, and then self-ligated at 20 °C, overnight, using T4 DNA ligase, resulting in pAMX4. pAMX4 was transformed into *E. coli* ET12567 and then mobilized into *S. coelicolor* M1154 through *E. coli-Streptomyces* tri-parental mating^34^ using *E. coli* ET12567/pR9406^47^ as a helper strain to generate the GPAHex production chassis, *S. coelicolor* M1154/pAMX4.

### Construction of the copy number control vector pCGW

Based on the original pCAP03-aac(3)IV capture vector^30^, pCGW was designed by replacing the SuperCos I derived region with a copy number control region (*oriV-ori2-repE-incC-sopABC*) from pBAC-lacZ. Primers strep-F/R and yeast-F/R were used to amplify the *Streptomyces* and yeast elements from pCAP03-aac(3)IV, respectively. Since there is a *Nde*I site in the *repE* gene in the copy number control cassette, overlap-extension PCR was used to mutate this *Nde*I site. Primers oriVS-sop-F and repE-mNdeI-R were used to amplify one half of the copy number control cassette from pBAC-lacZ, while primers repE-mNdeI-F and oriVS-sop-R were used to amplify the other half. Then primers oriVS-sop-F/R were used to stitch the two fragments, resulting in the mutated copy number control cassette, which was further assembled into pCGW with previously amplified *Streptomyces* and yeast elements through Gibson assembly^48^.

### TAR cloning and heterologous expression of the GPA BGCs

GPA BGCs were identified by analyzing the genome sequences using antiSMASH 5.0 before TAR cloning. TAR cloning was performed by following the standard protocol of direct cloning of NP BGCs^49^ using pCGW instead of pCAP03-acc(3)IV. pCGW was treated with *Nde*I and *Xho*I followed by introduction of gBlocks bearing capture hooks into pCGW by Gibson assembly. gBlocks for capturing corbomycin, GP1416, and GP6738 BGCs are listed in Supplementary Table 1. pCGW bearing capture hooks were linearized by *Pme*I and used to transform yeast spheroplasts. Genomic DNA from *Streptomyces* sp. WAC01529, *Amycolatopsis* sp. WAC01416, and *Streptomyces* sp. WAC06738 were isolated using the salting out procedure followed by RNase A treatment^34^, and then digested with *BstZ*17I/*Hpa*I, *BstZ*17I, and *Hind*III, respectively. Linearized pCGW-derived capture plasmids (500 ng) and digested genomic DNA (2 μg) were mixed and co-transformed into *S. cerevisiae* VL6-48N spheroplasts and plated onto SD-Trp-5-FOA selection medium for growing 3–5 d^49^. Plasmid DNA was extracted from yeast colonies using the alkaline lysis method for PCR screening. Positive hits were re-transformed into *E. coli* EPI300 cells using electroporation and then confirmed by restriction mapping. Sequence confirmed constructs were isolated and transformed into *E. coli* ET12567 and conjugated into *S. coelicolor* M1154 and *S. coelicolor* M1154/pAMX4, through tri-parental mating as described above.

### Determine of the integration site of GPA BGCs

*S. coelicolor* M1154/pAMX4/pGP1416, *S. coelicolor* M1154/pAMX4/pGP1529, and *S. coelicolor* M1154/pAMX4/pGP6738 strains were grown at 30 °C, 250 rpm, in TSBY medium (supplemented with 50 μg/mL kanamycin and 25 μg/mL thiostrepton) to mid-log phase, and then harvested for genomic DNA preparation using the salting out procedure^34^. Illumina MiSeq sequencing (300 bp, paired end reads) was performed by the McMaster Genomics Facility in the Farncombe Institute at McMaster University. The high complexity of the *crb* and GP6738 BGCs results in an inability to resolve the chromosomal integration loci through genome sequencing, so PCR diagnostics were used instead for these cases. crb-up-dF/dR, crb-m-dF/dR, and crb-dw-dF/dR primers were used to confirm the integration of *crb* BGC into the chromosome through homologous recombination between the *attL-int* regions. Similarly, GP6738-up-dF/dR, GP6738-m-dF/dR, and GP6738-dw-dF/dR primers were used to confirm the integration of GP6738 BGC into the chromosome at the same site.

### Deletion of the acyltransferase coding gene on pGP1416

*orf22* on pGP1416 was deleted using λ-red mediated PCR targeting as shown in Supplementary Fig. 8^27^. Δorf22_1416_-F/R primers were used to amplify the *aac(3)IV* resistance cassette from pCGW. The *aac(3)IV* cassette was transformed into *E. coli* BW25113/pKD46/pGP1416 through electroporation, resulting in pGP1416Δ*orf22*::*aac(3)IV*. pGP1416Δ*orf22*::*aac(3)IV* was then digested with *Pac*I overnight at 37 °C to remove the *aac(3)IV* cassette. *Pac*I digested pGP1416Δ*orf22*::*aac(3)IV* plasmid was precipitated and self-ligated at 20 °C using T4 DNA ligase to generate a scarless in-frame deletion mutant of *orf22*, resulting in pGP1416Δ*orf22*. pGP1416Δ*orf22* was then conjugated from *E. coli* ET12567 to *S. coelicolor* M1154/pAMX4 through tri-parental mating to produce deacyl-GP1416.

### Metabolic analysis of corbomycin and GP6738

*Streptomyces* production strains were inoculated into 5 mL TSBY medium supplemented with the required selection antibiotics (50 μg/mL kanamycin for *S. coelicolor* M1154 exconjugants, 50 μg/mL kanamycin and 25 μg/mL thiostrepton for *S. coelicolor* M1154/pAMX4 exconjugants), and then incubated at 30 °C, 250 rpm for 48 h. A 1 mL seed culture of each strain was inoculated into 50 mL SAM medium in 250 mL flasks, and incubated at 30 °C, 250 rpm for 6 days. The fermentation broth was harvested and lyophilized, followed by washing with 50% MeOH (5 mL) ×3 to remove hydrophilic impurities. Remaining insoluble material was extracted with DMSO (5 mL for corbomycin producing strains and 10 mL for GP6738 producing strains) and subjected to HPLC on a Hypersil GOLD aQ C18 Polar Endcapped HPLC column (175 Å, 3 µm, 4.6 mm × 150 mm, Thermo Scientific 25303-154630). Gradient conditions for corbomycin detection are as follows: *t* = 0–1 min, 5% solvent B; *t* = 20–22 min, 100% solvent B; *t* = 23–27 min, 5% solvent B (solvent A: 0.1 % *v/v* trifluoroacetic acid [TFA] in water and solvent B: 0.1 % *v/v* TFA in acetonitrile [ACN]); flow rate 1 mL/min, 40 °C. Gradient conditions for GP6738 detection are as follows: *t* = 0–2 min, 0% solvent B; *t* = 10 min, 50% solvent B; *t* = 15 min, 70% solvent B; *t* = 16–20 min, 0% solvent B (solvent A: 0.01% *v/v* NH_4_OH in water [pH 9.6] and solvent B: ACN); flow rate 1 mL/min, 40 °C. NH_4_OH was also added to the GP6738 samples (0.01% (*v/v*) NH_4_OH final) before injection.

### Metabolic analysis of GP1416 and deacyl-GP1416

*Streptomyces* strains were cultured as described above. The fermentation broth was treated and extracted using AffiGel 10-d-Ala-d-Ala affinity column^24^. Supernatant cultures (20 mL) were mixed with AffiGel 10-d-Ala-d-Ala resin (2 mL) at room temperature for 1 h and then packed into a small column (5 mL) running with 10 mL of 0.02 M phosphate buffer (pH 7.0), followed by washing with 10 mL 0.5 M ammonium acetate (pH 7.8) and 10 mL 10% ACN. GPA can be eluted from the AffiGel 10-d-Ala-d-Ala resin by eluting with 20 mL 50% ACN (0.1 M ammonium hydroxide), followed by adjusting the pH to 7.0–7.5 using formic acid, and then lyophilized for further HPLC and HPLC-MS analysis. Partially purified extracts were analyzed by HPLC using Symmetry Shield RP 8 Column (100 Å, 3.5 µm, 4.6 mm × 150 mm column, Waters, WAT094269) using the following gradient: *t* = 0 min, 5% solvent B; *t* = 2 min, 23% solvent B; *t* = 27 min, 30% solvent B; *t* = 32–35 min, 100% solvent B; *t* = 35.1–40 min, 5% solvent B (solvent A: 0.1% *v/v* TFA in water and solvent B: 0.1% *v/v* TFA in ACN); flow rate 1 mL/min, 40 °C. Deacyl-GP1416 was analyzed using the following gradient: *t* = 0–1 min, 0% solvent B; *t* = 20 min, 100% solvent B; *t* = 20.1–25 min, 0% solvent B (solvent A: 0.1% *v/v* TFA in water and solvent B: 0.1% *v/v* TFA in ACN); flow rate 1 mL/min, 40 °C. Deacyl-GP1416 extract was treated with 0.4% (*v/v*) formic acid for 1 h at 37 °C before injection.

### Purification of deacyl-GP1416

*Streptomyces* strains were grown as described above. The culture supernatant was treated with 5% (*w/v*) Diaion HP-20 resin (Sigma-Aldrich), followed by elution with 10% methanol and 50% ACN using column chromatography. The 50% ACN fraction was applied to a Sephadex LH20 (Sigma-Aldrich) column using 50% ACN as the running solvent. Active fractions were collected and further purified by HPLC on an Agilent semi-prep column (Zorbax Extend C-18, 21.2 × 100 mm, 5 μm) using a linear gradient from 5 to 10% solvent B (solvent A, 0.1% *v/v* formic acid in water and solvent B, 0.1% *v/v* formic acid in ACN) over 10 mins to yield pure compound.

### Overexpression of the regulators in GP6738 BGC

strR_6738_-F/R and lmbU_6738_-F/R primers were used to amplify *strR* and *lmbU* genes from *S*. sp. WAC06738. The two amplicons were then assembled into *Nde*I/*Avr*II treated pIJ10257^50^ plasmid using Gibson assembly, resulting in pIJ10257-sl. Error-free pIJ10257-sl plasmid was conjugated into *S. coelicolor* M1154/pGP6738 and *S. coelicolor* M1154/pAMX4/pGP6738 through tri-parental mating as described above.

### Purification of GP6738

*S. coelicolor* M1154/pAMX4/pGP6738/pIJ10257-sl was inoculated in TSBY medium (supplemented with 50 μg/mL kanamycin, 25 μg/mL thiostrepton, and 50 μg/mL hygromycin) and grown at 30 °C, 250 rpm for 48–60 h. Then 1 mL of the seed culture was inoculated into 50 mL SAM medium in 250 mL flasks, and incubated at 30 °C, 250 rpm for 6 days. 150 mL of SAM culture was collected and lyophilized. The dried material was washed with 50% MeOH (4×) and extracted with DMSO (3×), resulting in ~70 mg pure GP6738. GP6738 was dissolved into *d6*-DMSO (20 mg/mL) and analyzed by 1D and 2D NMR recording on a Bruker AVIII 700 MHz instrument equipped with a cryoprobe. GP6738 was analyzed by high-resolution mass spectrometry (HRMS) using an Agilent 6550 iFunnel Q-TOF mass spectrometry with an inline Agilent 1290 HPLC system using electrospray ionization in positive mode, and mass scan range from 50–2000 Da. MS/MS spectrum of GP6738 was recorded on the same system using a collision-induced dissociation (CID) energy of 50 V. A Symmetry Shield RP 8 column (100 Å, 3.5 µm, 4.6 mm × 150 mm column, Waters, WAT094269) was used with the following gradient: *t* = 0–1 min, 5% solvent B; *t* = 20–22 min, 100% solvent B; *t* = 23–30 min, 5% solvent B (solvent A: 0.1% *v/v* formic acid in water and solvent B: 0.1% *v/v* formic acid in ACN); flow rate 0.6 mL/min, 40 °C.

### RNA isolation and RT-PCR

*Streptomyces* strains were inoculated into 50 mL SAM medium in 250 mL flasks using 1% (*v/v*) inoculum from TSBY seed cultures, and mycelia were harvested at early-stationary phase (20 h) and late-stationary phase (48 h). Cells were lysed by bead beating with 4 mm glass beads in 5 mL TRIzol reagent (Invitrogen), and RNA was extracted and purified using the PureLink RNA Mini Kit (Invitrogen) according to the manufacturer’s recommendations. cDNA synthesis was performed using Maxima H Minus First Strand cDNA synthesis Kit (Thermo Fisher Scientific) after dsDNase (Thermo Fisher Scientific) treatment, followed by RT-PCR quantification on a BioRad CFX96 real time system using PowerUp SYBR Green Master Mix (Applied Biosystems). Primers targeting genes of interest were designed (Supplementary Data 2) and 90–100% efficiency was confirmed before quantitation. Analysis was performed on biological triplicates and the fold change of gene expression was calculated by normalizing to *hrdB* using the ΔCt method.

### Phylogenetic analysis of P450s

Protein sequences were obtained from the NCBI database and aligned with Muscle using default settings in MEGA X. A maximum likelihood phylogeny was created by MEGA X^51^ with 100 bootstrap replicates using the WAG substitution model with empirical amino acid frequencies (+F), gamma-distributed rates invariant sites (G + I), complete deletion of gaps, and the Nearest-Neighbor-Interchange (NNI) tree search method. The consensus tree was re-rooted to preserve the monophyletic OxyB.

### Antibiotic susceptibility testing

Antibiotic susceptibility was tested using standard procedures in Mueller-Hinton Broth for most strains. *Enterococcus* strains were grown in Brain Heart Infusion media (BHI; BD Biosciences). *Streptomyces* MICs were determined in Bennett’s media (10 g potato starch, 2 g casamino acids, 1.8 g yeast extract, 0.2 g KCl, 0.2 g MgSO_4_·7H_2_O, 0.24 g NaNO_3_, 4 mg FeSO_4_·7H_2_O, 1 L ddH_2_O, pH 6.8).

### Cell lysis assay

Early exponential phase *B. subtilis* 168 (OD_600_ ~0.25) grown in LB media was dispensed 100 µL per well into a round bottom 96 well plate. Antibiotics were supplemented to the appropriate concentration, and where appropriate lytic agents were added (50 µg/mL fosfomycin, 100 µg/mL ampicillin, 100 mM sodium azide). The plate was covered with a clear, breathable film and OD_600_ was monitored on a Tecan sunrise microplate reader at 37 °Cwith shaking.

### Peptidoglycan autolysin digestion

The muramidase mutanolysin was purchased from Sigma. The glucosaminidase domain from LytD was cloned from *B. subtilis* 168 into pET28 between *Nco*I and *Xho*I restriction sites for introduction of C-term 6× His. Construct was expressed from *E. coli* BL21(DE3) pLysS by inoculating 1 L of LB media 1:50 from an overnight culture, then growing at 37 °C until OD_600_ reached 0.6, at which time the cells were induced with 1 mM IPTG and grown for 18 h at 16 °C. Native LytD-6× His was purified by Ni-NTA affinity chromatography and cation exchange chromatography^14^. Protein purity was >95% as assessed by SDS-PAGE analysis.

*B. subtilis* peptidoglycan for binding and digestion assays was prepared by harvesting the cells (OD_600_ 0.6–0.7), boiled in 4% SDS, and washed and sonicated to break up sacculi, followed by α-amylase and DNase treatment, and digested with pronase overnight^14^. The peptidoglycan was boiled in 2% SDS again, washed, and teichoic acids were hydrolysed with 1 M HCl. The peptidoglycan was washed with water to pH 6.0 and lyophilized to use. Peptidoglycan was digested under optimized conditions with either mutanolysin (20 µg/mL, 1 mg/mL PG, 20 mM sodium acetate, pH 6.5) or LytD (10 µg/mL, 1 mg/mL PG, 200 mM NaCl, 50 mM MES-NaOH, pH 5.5). 100 µL of peptidoglycan in buffer, as indicated, was preincubated with either GP6738 or a corresponding volume of DMSO with shaking for 30 minutes, then dispensed into a round bottom 96 well plate and enzyme added. The plate was covered with a clear, breathable film to prevent evaporation, and OD_600_ was tracked on a Tecan sunrise microplate reader shaking at 37 °C. Results show the average and standard deviation of triplicate wells.

### Peptidoglycan binding assay

To assess peptidoglycan binding, GP6738 was diluted to 0.1 mg/mL in water with 20% (*v/v*) DMSO to aid GP6738 solubility. For each 100 μL binding assay, *B. subtilis* peptidoglycan was added to 0 mg/mL, 2.5 mg/mL, 5 mg/mL, 10 mg/mL or 20 mg/mL for 0, 25:1, 50:1, 100:1 and 200:1 *w/w* ratios, respectively. Mixtures were incubated for 1–2 h at 37 °C, then centrifuged for 10 min to thoroughly pellet insoluble material, and the supernatant was removed for analysis. Samples were prepared for HPLC analysis by the addition of NH_4_OH to 0.01% (*v/v*), and 50 µL was injected on the HPLC. Analysis was performed using a Thermo Hypersil GOLD aQ C18 4.6 × 150 mm column and the following gradient at 0.8 mL/min, 40 °C: *t* = 0–2 min 0% solvent B, *t* = 10–13 min 65% solvent B, *t* = 14 min 0% solvent B (solvent A: 0.01% *v/v* NH_4_OH in water [pH 9.6] and solvent B: ACN).

### Statistical analysis

Statistical analysis of compound production quantified by LC-MS and HPLC and gene-expression quantified by RT-PCR was performed using GraphPad Prism v. 7 in every case. For corbomycin and GP6738 quantitation, statistical significance was assessed by unpaired two-sided Student’s *t*-test (*n* = 3) or one-way ANOVA with Tukey’s post hoc analysis (*n* = 3) as described in the figure legends. For gene expression analysis, two-way ANOVA with Tukey’s post hoc analysis (*n* = 3) was performed as described in the figure legends. *P* values <0.05 were considered as statistically significant. All results are representative of two independent experiments.

### Reporting summary

Further information on research design is available in the Nature Research Reporting Summary linked to this article.

## Supplementary information

Supplementary Information

Reporting Summary

Description of Additional Supplementary Files

Supplementary Data 1–2

## Data Availability

Data supporting the findings of this work are available within the paper and its Supplementary Information files. A reporting summary for this article is available as a Supplementary Information file. The datasets and materials generated and analyzed during the current study are available from the corresponding author upon request. Genome sequences of *Streptomyces* sp. WAC01529, *S*. sp. WAC06738, *Amycolatopsis* sp. WAC01416, *S. coelicolor* M1154/pAMX4, and *S. coelicolor* M1154/pAMX4/pGP1416 are available in GenBank with accession numbers NZ_CP029617.1, NZ_CP029618.1, NZ_QHHX00000000.1, JAATOK000000000.1, and CP050522.1, respectively. Concatenated TIGRFAM core-gene phylogeny of species with GPA BGCs shown in Supplementary Fig. 19 is available at GitHub [http://github.com/waglecn/GPA_evolution.git]. Source data are provided with this paper.

## Source data

Source data
