# Supplementary material for: GPAHex-A synthetic biology platform for Type IV–V glycopeptide antibiotic production and discovery
Source: Nat Commun. 2020 Oct 16;11:5232. doi: 10.1038/s41467-020-19138-5 (PMC7567792; doi:10.1038/s41467-020-19138-5)
Supplement: Supplementary file 1 — Supplementary Information [file 41467_2020_19138_MOESM1_ESM.pdf]

GPAHex-A synthetic biology platform for TypeIV-V glycopeptide antibiotic production and discovery

Xu *et al.*

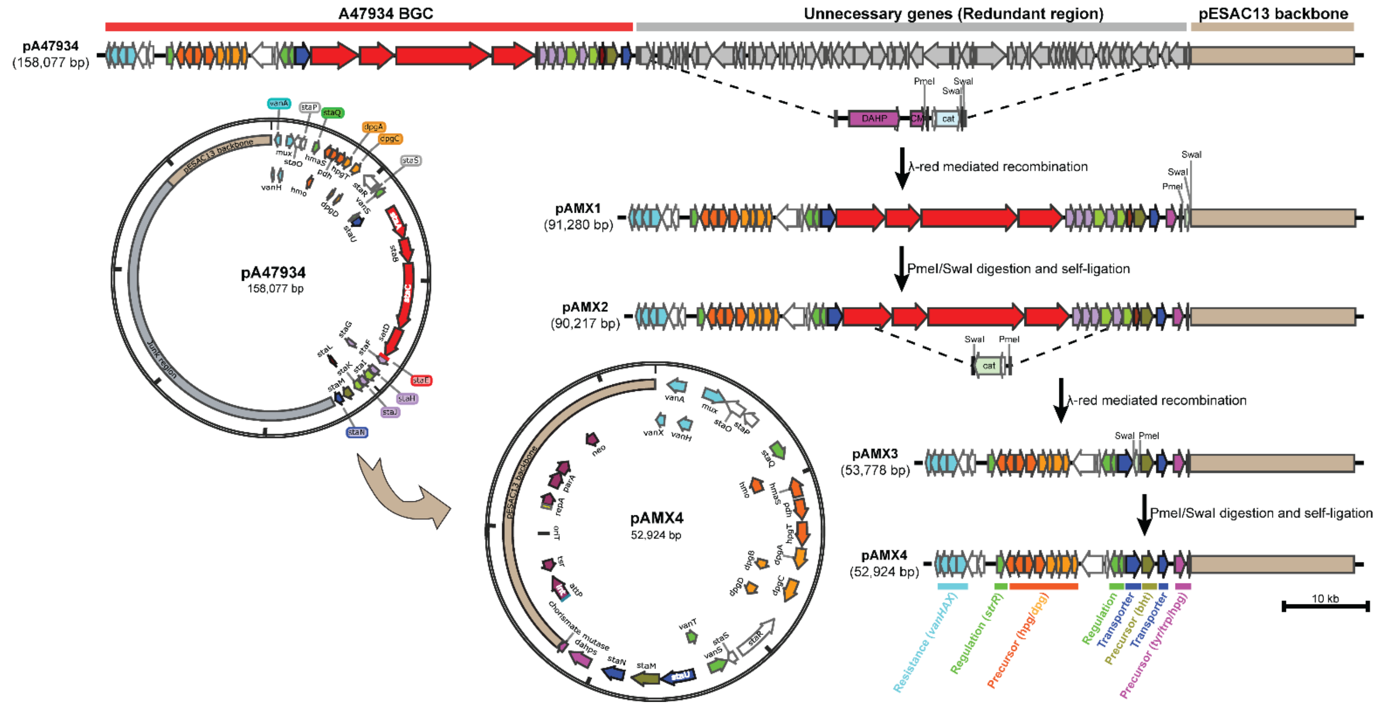

Supplementary Fig. 1. Schematic representation of the construction of pAMX4. pA47934 was determined by end sequencing from a previously identified construct from S. toyocaensis NRRL15009 PAC genomic library. pAMX1 was derived from pA47934 by deleting the region unrelated to A47934 biosynthesis and replacing it with a dual gene cassette bearing DAHP synthase and chorismate mutase encoding genes, which are gatekeeper genes of shikimate pathway for aromatic amino acids biosynthesis, amplified from S. toyocaensis NRRL15009 genome in situ. pAMX2 was derived from pAMX1 by further deletion of the NRPS scaffold genes and the tailoring genes (P450s, halogenase, and sulfotransferase encoding genes), while leaving behind the precursor supply genes for Hpg, Dpg, and Bht biosynthesis, glycopeptide resistance genes mux-vanHAX, and glycopeptide BGC-associated positive strR family regulator and transporter genes. Functional genes are color-coded as described in the main text.

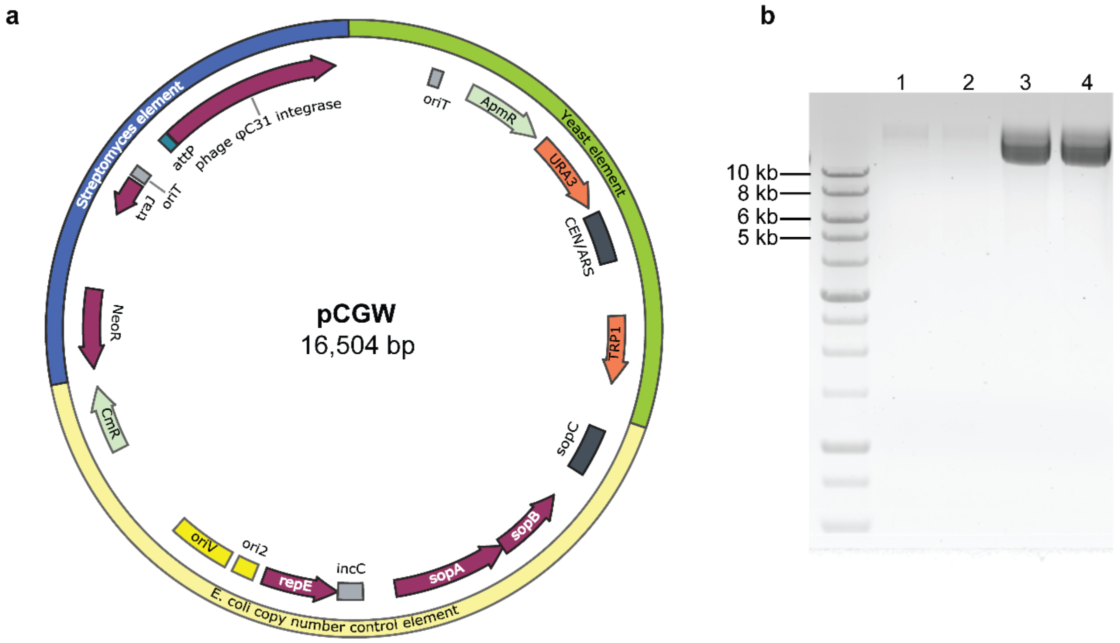

Supplementary Fig. 2. Copy number controlled plasmid pCGW for TAR cloning. **a**, Vector map of pCGW. pCGW was derived from pCAP03-aac(3)IV by replacing the Supercos I derived region with the copy number control element from pBAC-lacZ. **b**, Agarose gel of pCGW extracted from *E. coli* EPI300. Lanes 1 and 2 showing very faint bands represent the pCGW plasmid extracted from *E. coli* EPI300 without L-arabinose induction, while lanes 3 and 4 show the pCGW plasmid extracted from *E. coli* EPI300 with L-arabinose (1 mM) induction. Source data underlying Supplementary Figure 2b is provided as a Source Data file.

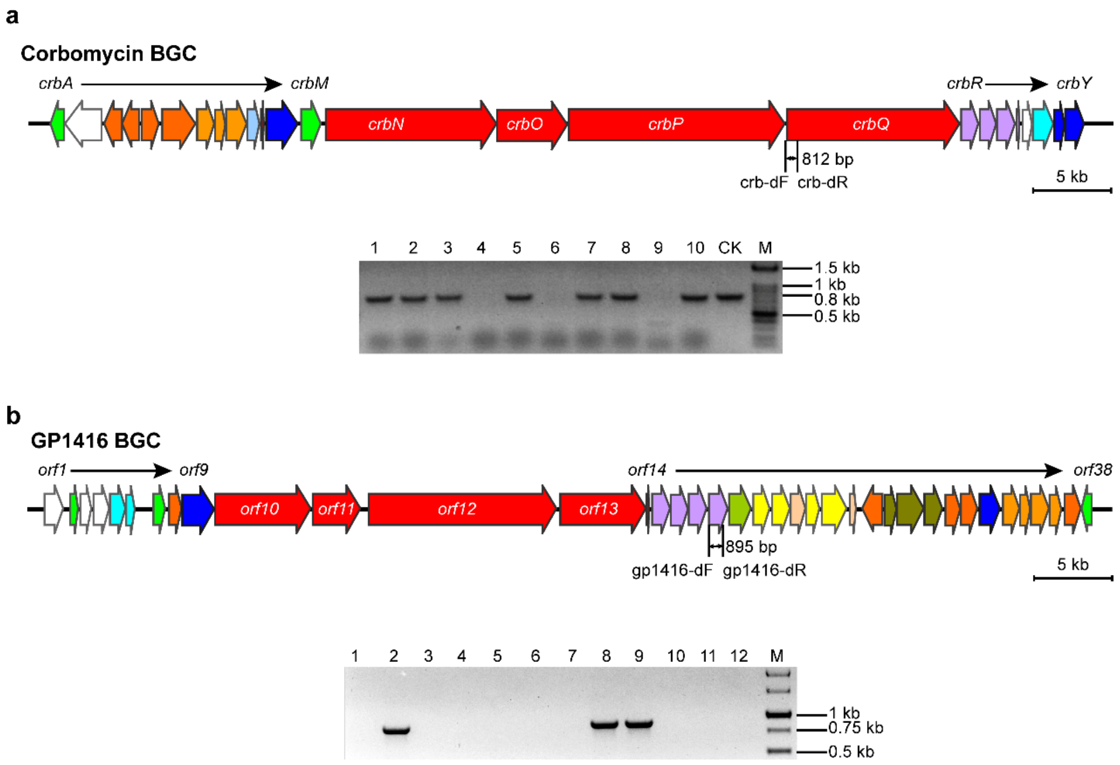

Supplementary Fig. 3. PCR screening of corbomycin and GP1416 BGC captured constructs using TAR cloning. TAR cloning efficiencies for capturing corbomycin BGC (**a**) and GP1416 BGC (**b**) were 70% (7/10) and 25% (3/12), respectively. CK indicates PCR product amplified from *S.* sp. WAC01529 genomic DNA. M represents 1 kb DNA ladder. Similar results (**a-b**) were obtained from two independent experiments. Source data are provided as a Source Data file.

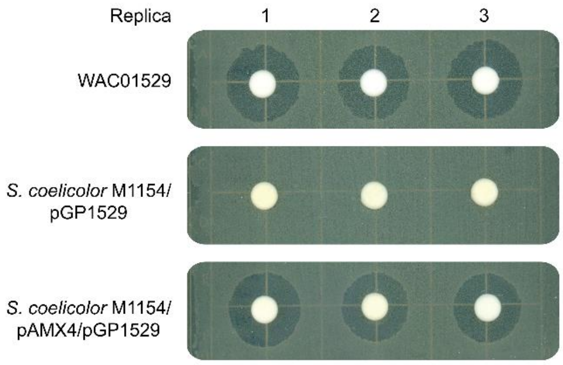

Supplementary Fig. 4. Bioassay of corbomycin heterologous expression extracts against *B. subtilis* 168. Crude extracts from *S. coelicolor* M1154/pGP1529 strain do not give zones of inhibition, while large zones are observed around crude extracts from *S. coelicolor* M1154/pAMX4/pGP1529. Zones of inhibition produced by the parental strain *S.* sp. WAC01529 are likely the result of uncharacterized compounds produced by this strain, as equivalent amounts of purified corbomycin as are present in these extracts do not give zones. Source data are provided as a Source Data file.

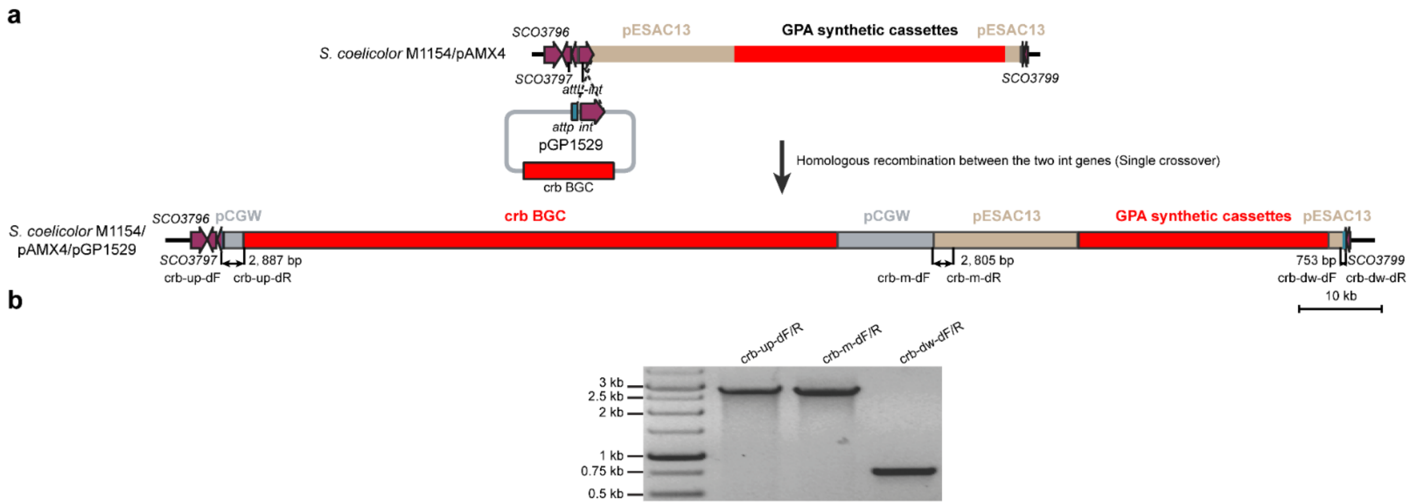

Spplementary Fig. 5. Integration of pGP1529 into the chromosome of *S. coelicolor* M1154/pAMX4. a, Schematic representation of homologous recombination mediated insertion of pGP1529 into *S. coelicolor* M1154/pAMX4 chromosome at the *attL-int* sequence. Three pairs of diagnostic primers crb-up-dF/R, crb-m-dF/R, and crb-dw-dF/R were used to confirm the recombination site through PCR. *S. coelicolor* genes and the recombination site *int* gene are shown as purple arrows. pAMX4 derived regions are shown in brown (pESAC13 backbone) and red (GPA synthetic cassettes), and pGP1529 derived regions are shown in gray (pCGW backbone) and red (*crb* BGC). **b**, Gel image of PCR products amplified from the genome of *S. coelicolor* M1154/pAMX4/pGP1529 using diagnostic primers. PCR products shown on the gel are consistent with their expected sizes. Similar results (**b**) were obtained from three independent experiments. Source data underlying Supplementary Figure 5b are provided as a Source Data file.

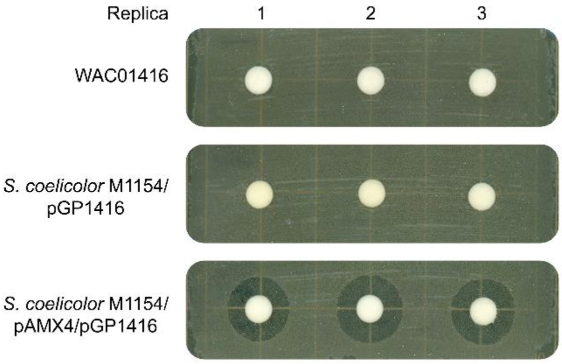

Supplementary Fig. 6. Bioassay of GP1416 heterologous expression extracts against *B. subtilis* 168. Neither the wild type strain *Amycolatopsis* sp. WAC01416 nor the heterologous expression strain *S. coelicolor* M1154/pGP1416 give zones of inhibition, while, the GPAHex strain bearing the GP1416 BGC produce large halos. Source data are provided as a Source Data file.

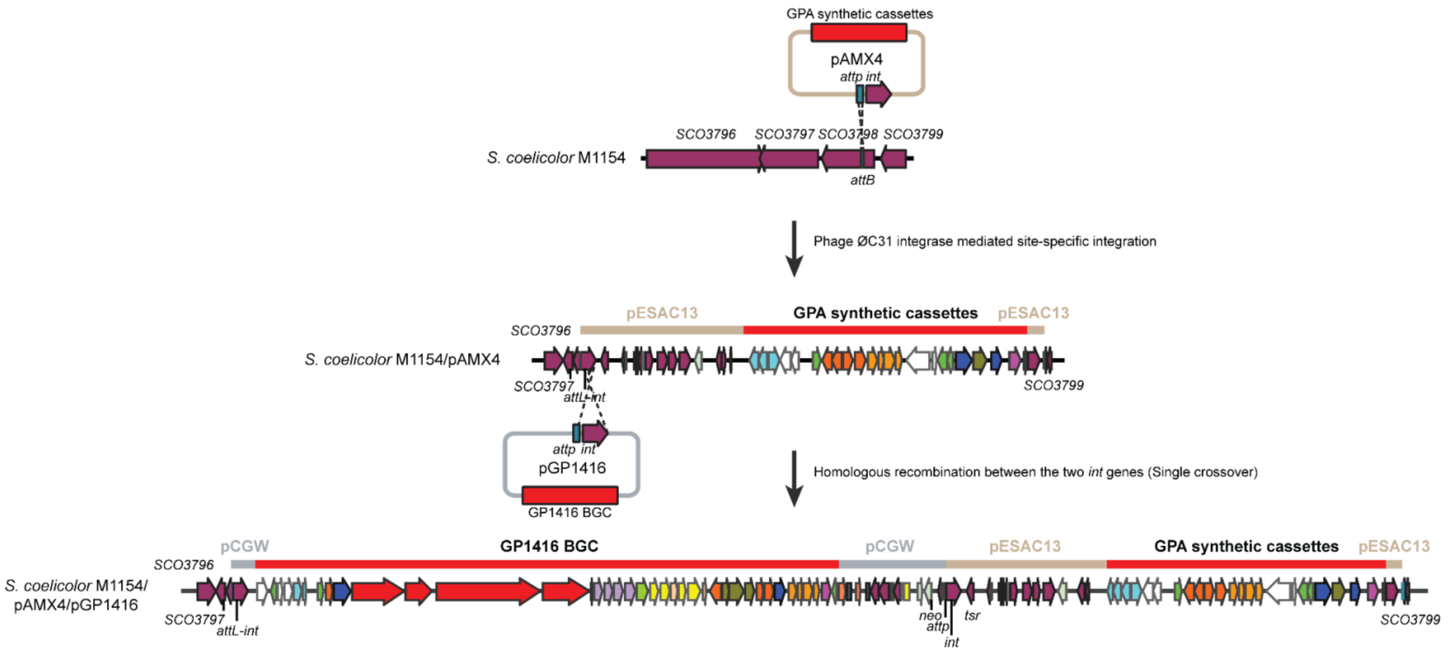

Supplementary Fig. 7. Integration of pGP1416 into the chromosome of S. coelicolor M1154/pAMX4 identified through genome sequencing. pAMX4 was inserted into the chromosome of *S. coelicolor* M1154 at $attB_{\Phi C31}$ site through phage ΦC31 integrase, resulting in the generation of the GPA production chassis, *S. coelicolor* M1154/pAMX4. pGP1416 bearing the BGC of GPA GP1416 was inserted into the chromosome of *S. coelicolor* M1154/pAMX4 through homologous recombination between the two *int* genes present in the backbones of pESAC13 and pCGW, resulting in the GP1416 production strain *S. coelicolor* M1154/pAMX4/pGP1416. This site of insertion was confirmed by Illumina and Nanopore genome sequencing.

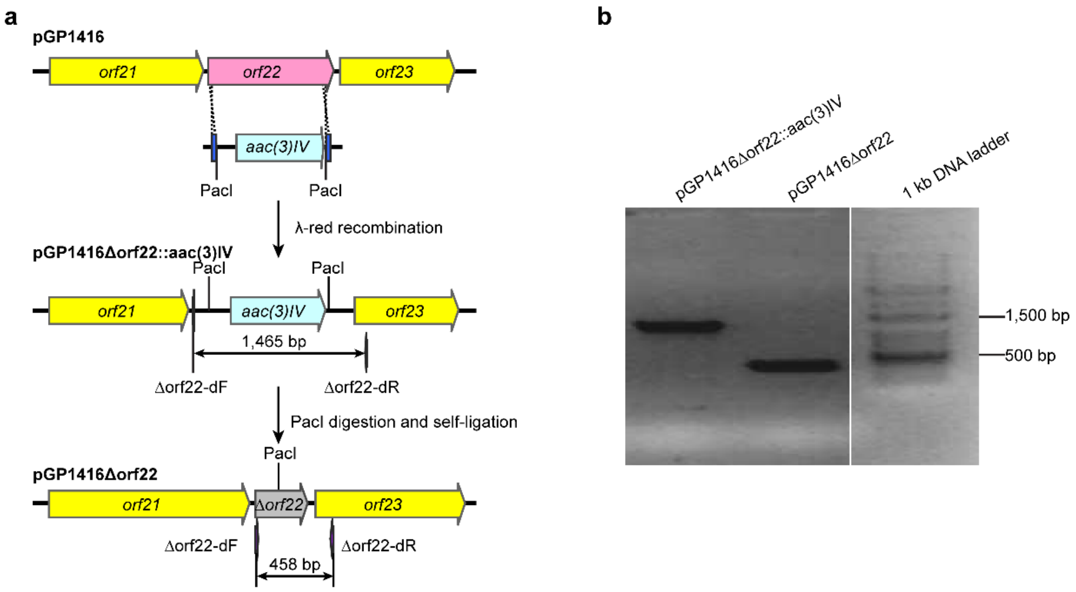

Supplementary Fig. 8. Deletion of the acyltransferase coding gene (*orf22*) on pGP1416. a, Schematic representation of the deletion of *orf22* on pGP1416 through λ-red mediated PCR targeting. **b**, Gel electrophoresis of PCR products amplified from pGP1416Δ*orf22*::*aac(3)IV* and pGP1416Δ*orf22*. Similar results (**b**) were obtained from two independent experiments. Source data underlying Supplementary Figure 8b are provided as a Source Data file.

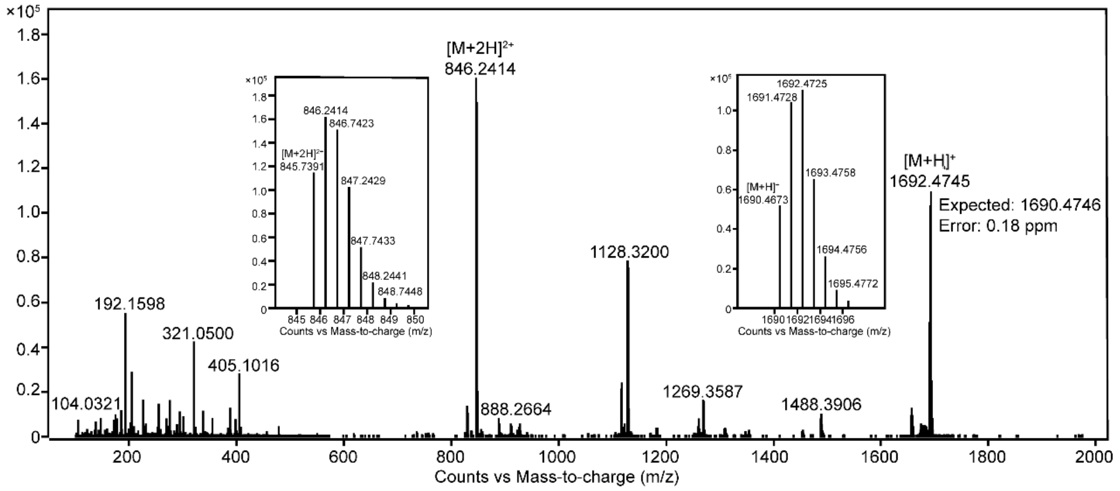

Supplementary Fig. 9. HR-MS of deacyl-GP1416 determined by HRESI-Q-TOF mass spectrometry. A singly charged ion of $[M+H]^+$=1690.4673 and a doubly charged ion of $[M+2H]^{2+}$=845.7391 were detected (error 0.18 ppm).

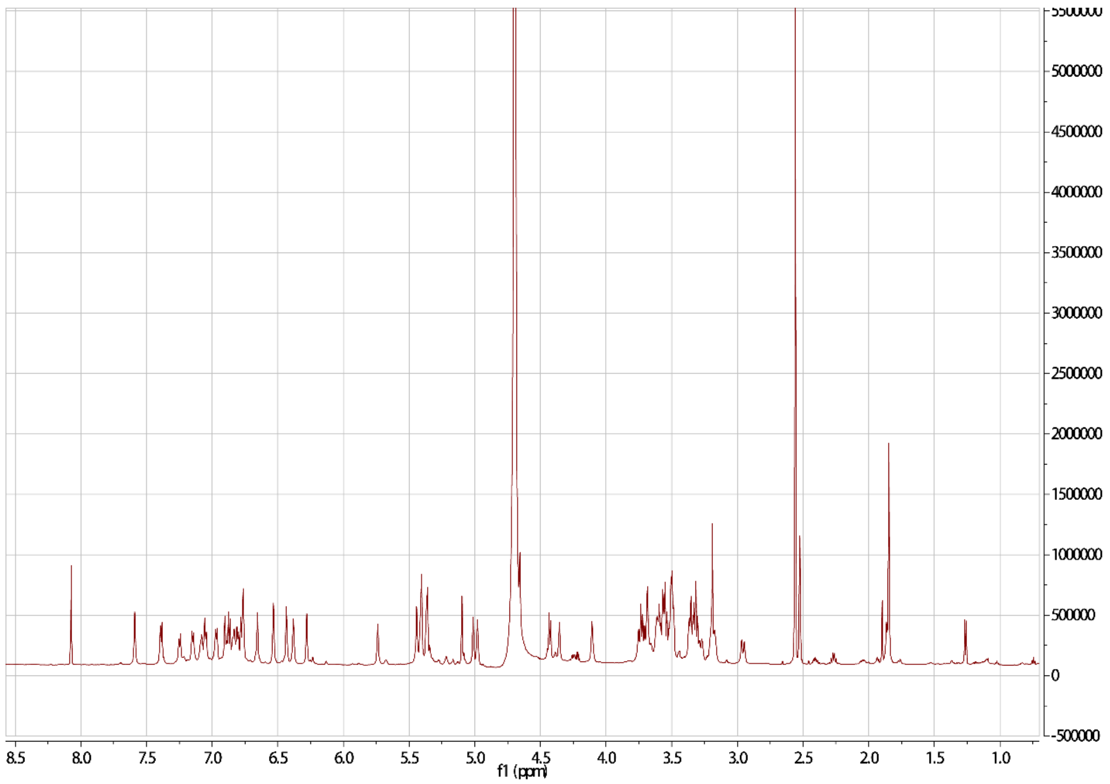

Supplementary Fig. 10. ^{1}H NMR spectrum of deacyl-GP1416 in D_2O.

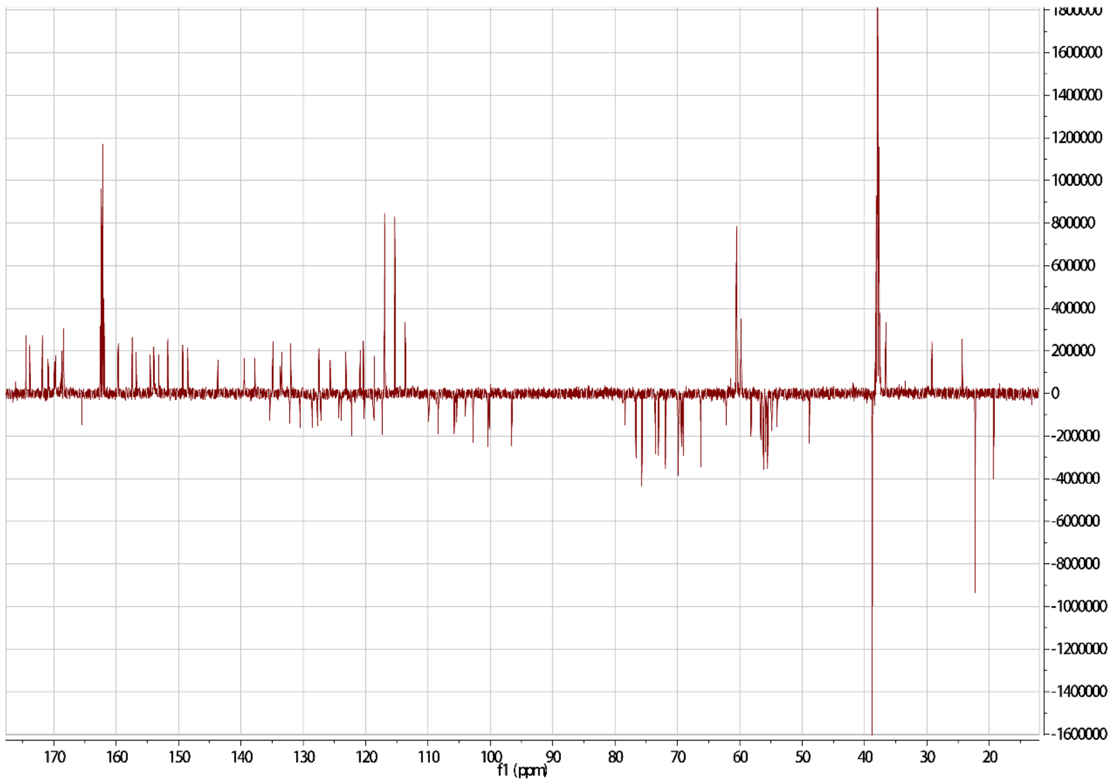

Supplementary Fig. 11. ^{13}C NMR spectrum of deacyl-GP1416 in D_2O.

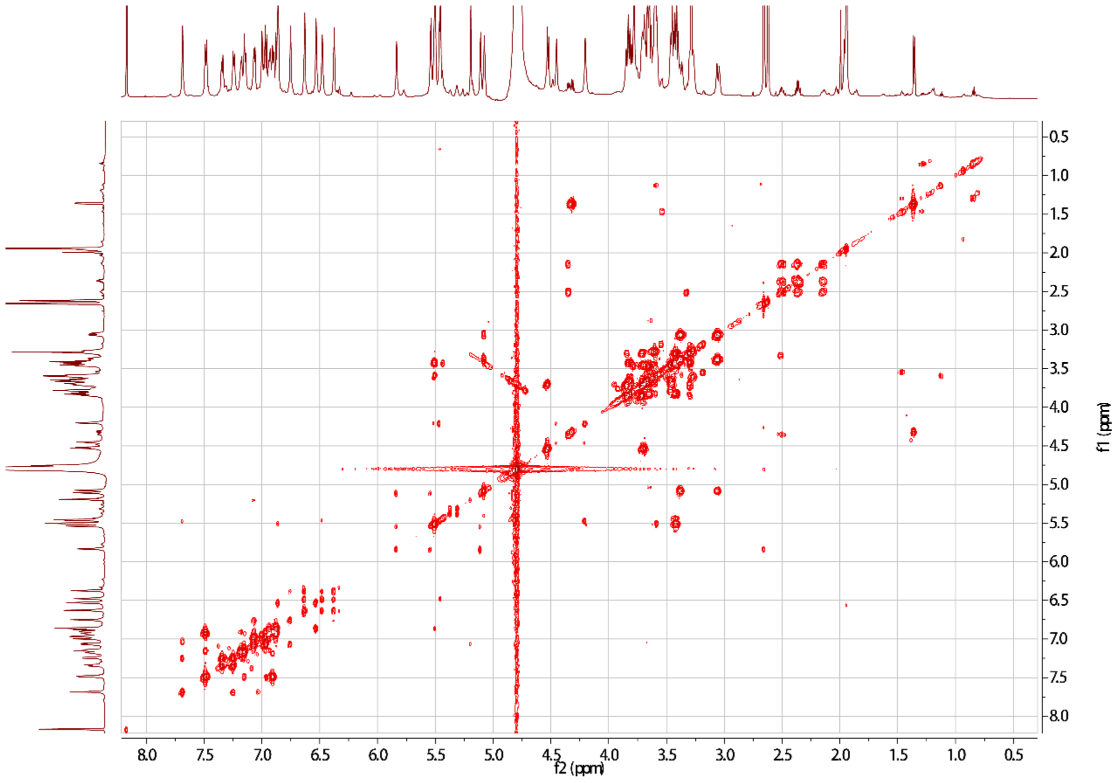

Supplementary Fig. 12. ^{1}H-^{1}H **COSY NMR spectrum of deacyl-GP1416 in** D_2O**.**

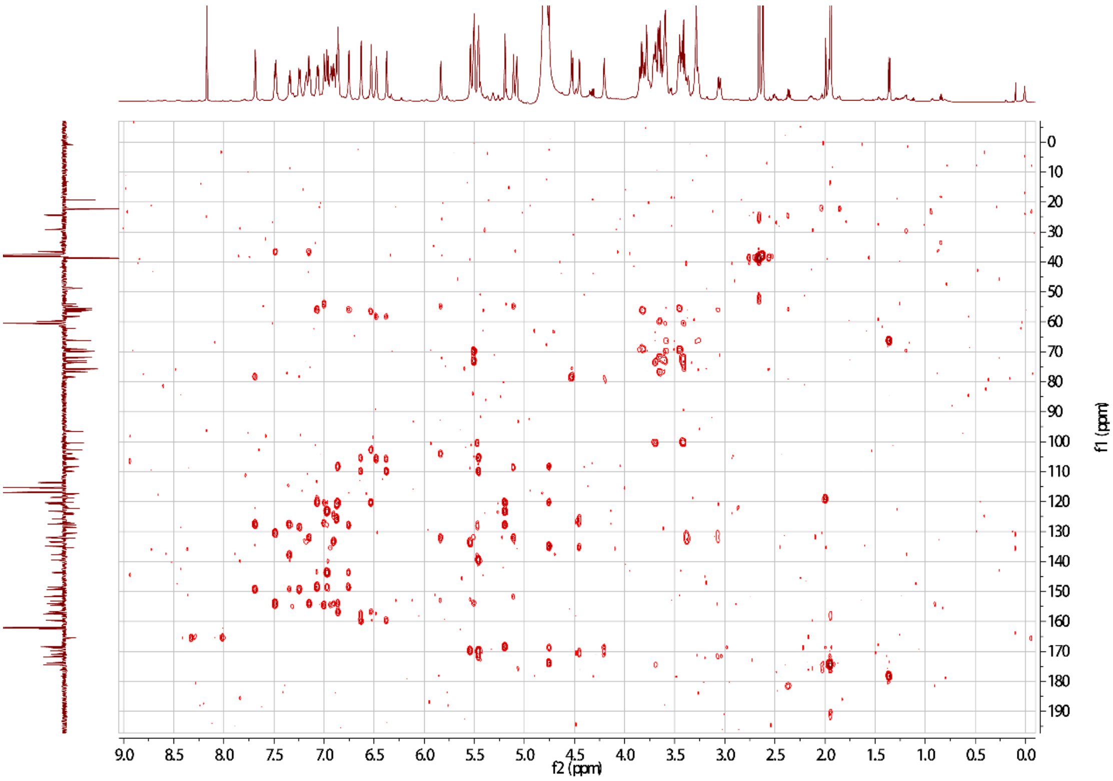

Supplementary Fig. 13. ^{1}H-^{13}C HMBC NMR spectrum of deacyl-GP1416 in D_2O.

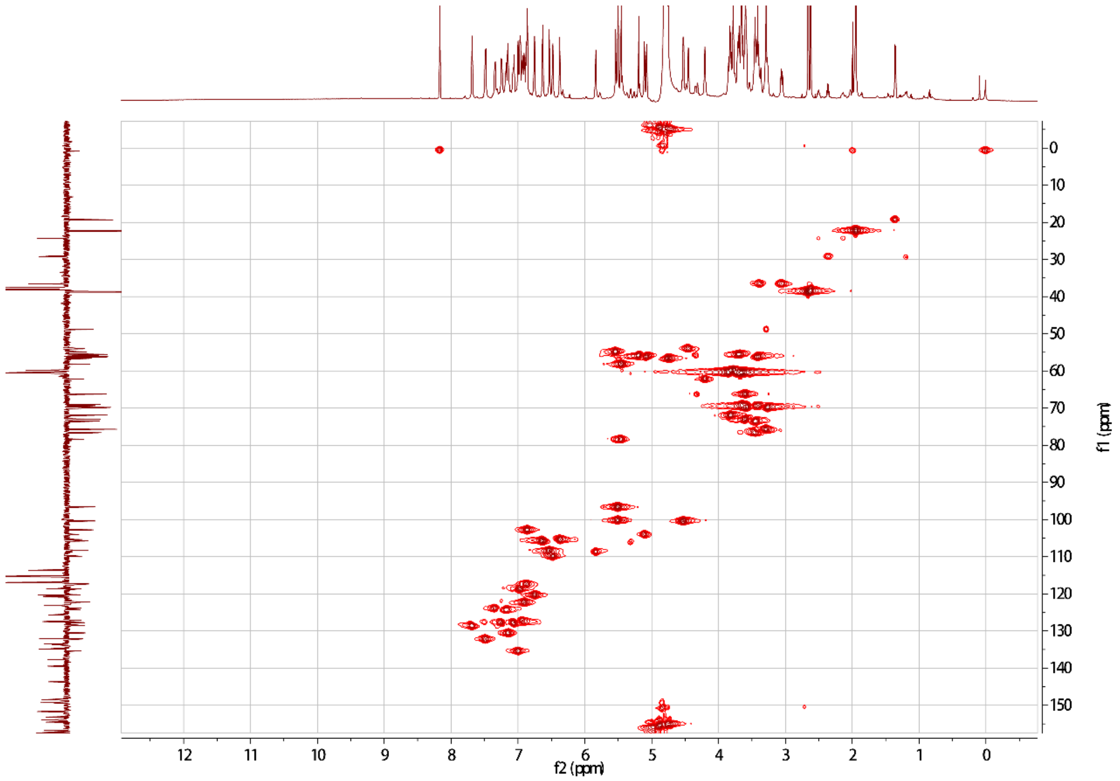

Supplementary Fig. 14. ^{1}H-^{13}C HSQC NMR spectrum of deacyl-GP1416 in D_2O.

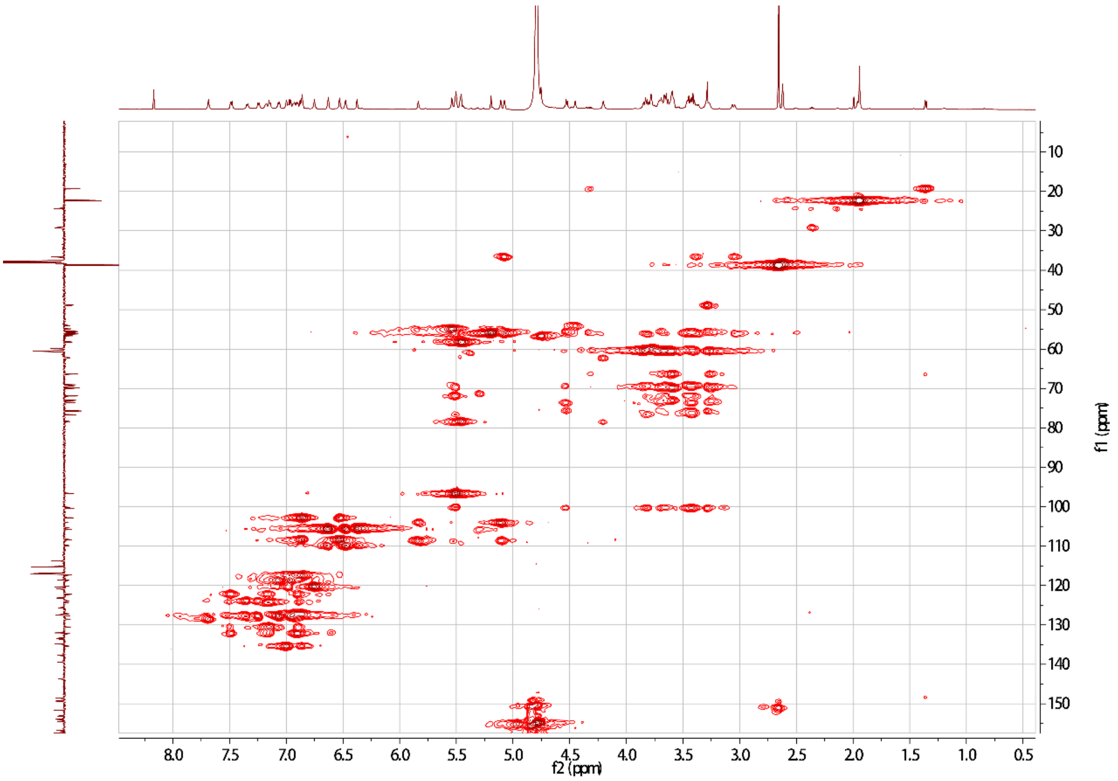

Supplementary Fig. 15. ^{1}H-^{13}C **TOCSY NMR spectrum of deacyl-GP1416 in** D_2O**.**

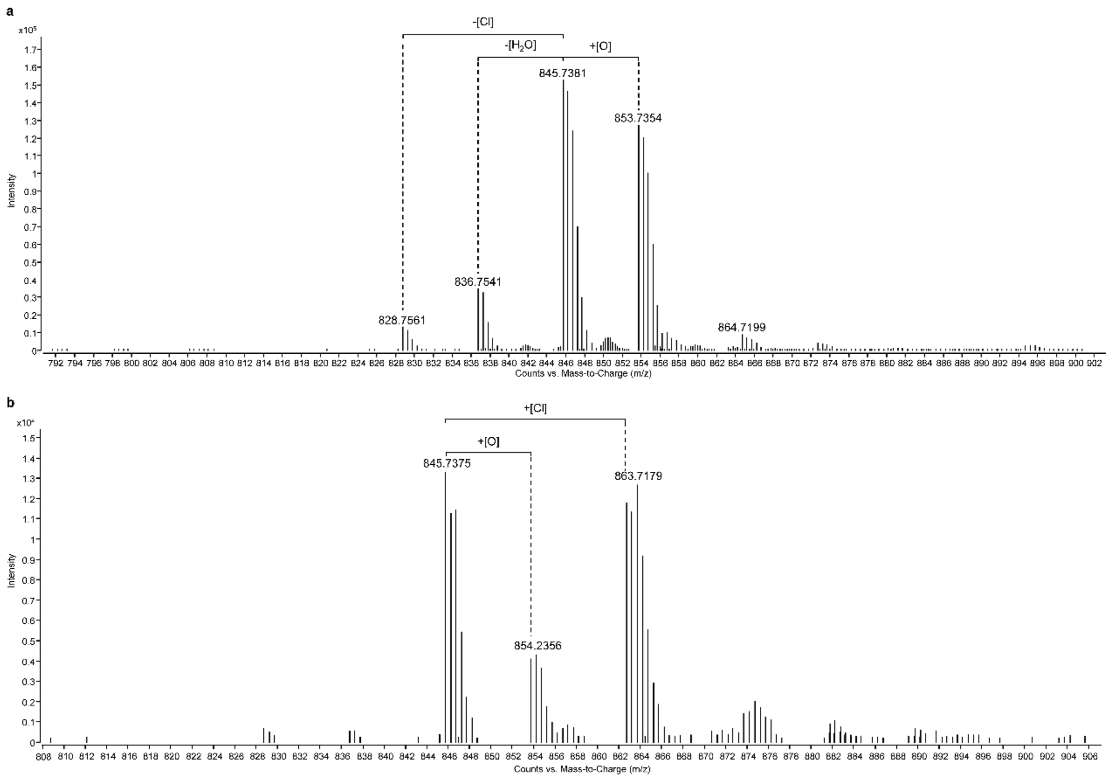

Supplementary Fig. 16. Mass spectra of deacyl-GP1416 analogs. The major analogs of deacyl-GP1416 are through chlorination and hydroxylation of the scaffold. All the mass signals shown above are doubly charged ion.

a

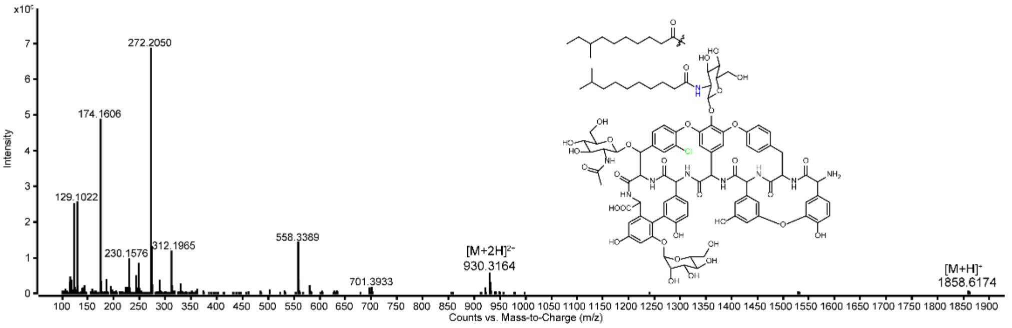
x10⁵
Intensity
272.2050
174.1606
129.1022
230.1576
312.1965
558.3389
701.3933
[M+2H]²⁺
930.3164
[M+H]⁺
1858.6174
Counts vs. Mass-to-Charge (m/z)

b

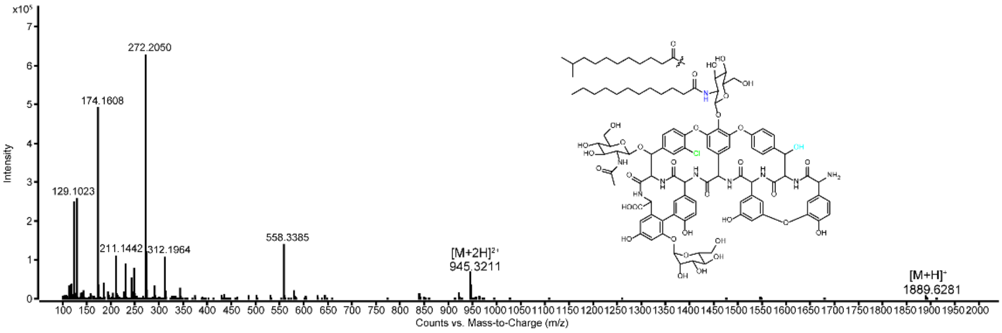
x10⁵
Intensity
272.2050
174.1608
129.1023
211.1442
312.1964
558.3385
[M+2H]²⁺
945.3211
[M+H]⁺
1889.6281
Counts vs. Mass-to-Charge (m/z)

c

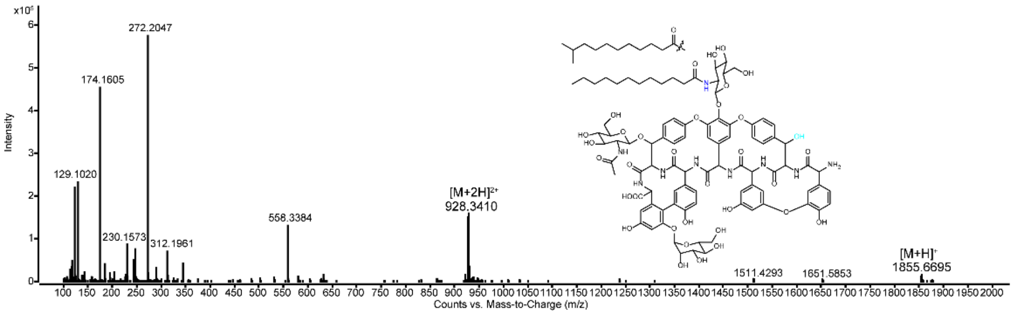
x10⁵
Intensity
272.2047
174.1605
129.1020
230.1573
312.1961
558.3384
[M+2H]²⁺
928.3410
1511.4293
1651.5853
[M+H]⁺
1855.6695
Counts vs. Mass-to-Charge (m/z)

d

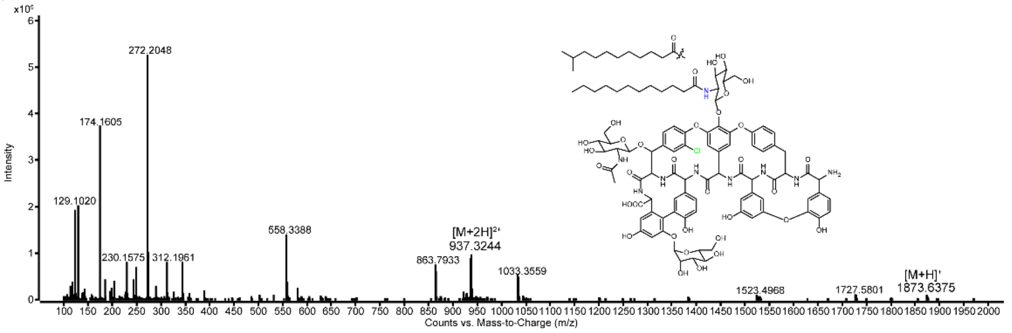
x10⁵
Intensity
272.2048
174.1605
129.1020
230.1575
312.1961
558.3388
863.7933
[M+2H]²⁺
937.3244
1033.3559
1523.4968
1727.5801
[M+H]⁺
1873.6375
Counts vs. Mass-to-Charge (m/z)

e

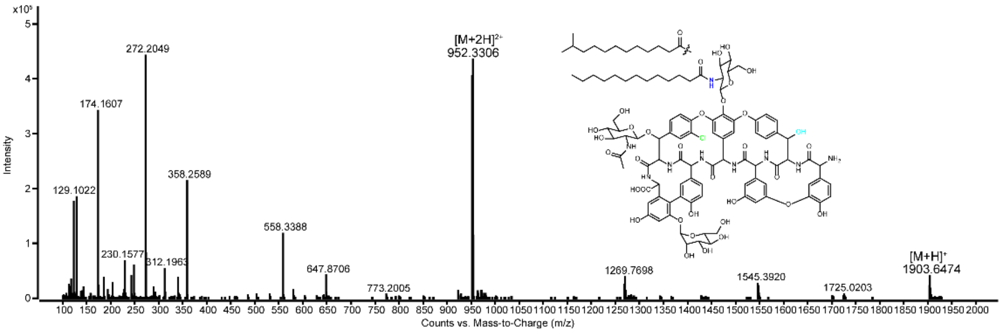
x10⁵
Intensity
[M+2H]²⁺
952.3306
272.2049
174.1607
129.1022
358.2589
230.1577
312.1963
558.3388
647.8706
773.2005
1269.7698
1545.3920
1725.0203
[M+H]⁺
1903.6474
Counts vs. Mass-to-Charge (m/z)

f

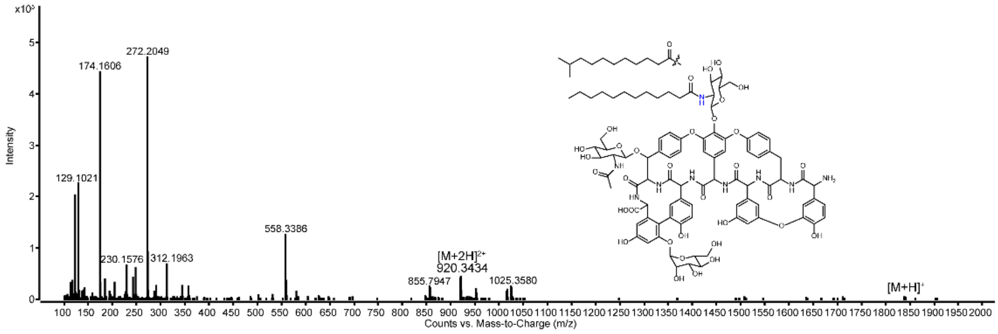
x10⁵
Intensity
272.2049
174.1606
129.1021
558.3386
230.1576
312.1963
[M+2H]²⁺
920.3434
855.7947
1025.3580
[M+H]⁺
Counts vs. Mass-to-Charge (m/z)

g

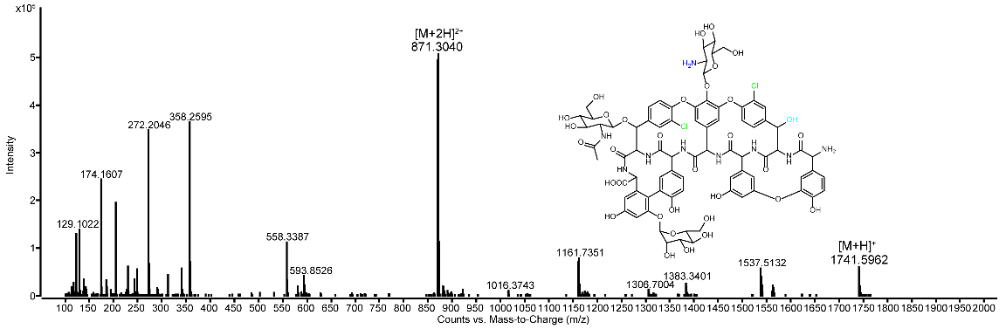
x10⁵
Intensity
[M+2H]²⁺
871.3040
272.2046
358.2595
174.1607
129.1022
558.3387
593.8526
1016.3743
1161.7351
1306.7004
1383.3401
1537.5132
[M+H]⁺
1741.5962
Counts vs. Mass-to-Charge (m/z)

h

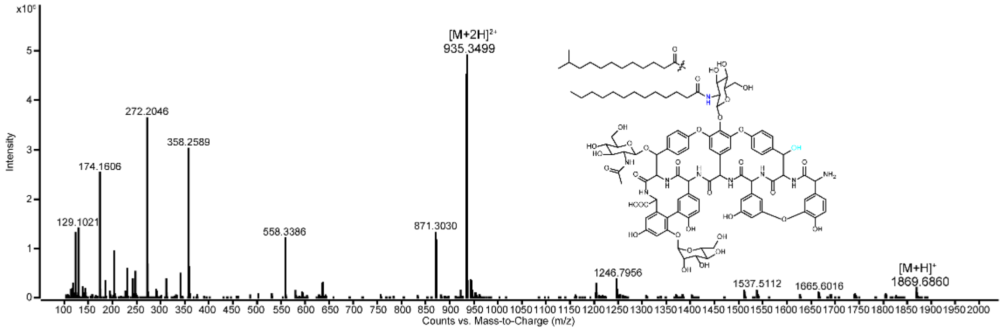
x10⁵
Intensity
[M+2H]²⁺
935.3499
272.2046
358.2589
174.1606
129.1021
558.3386
871.3030
1246.7956
1537.5112
1665.6016
[M+H]⁺
1869.6860
Counts vs. Mass-to-Charge (m/z)

i

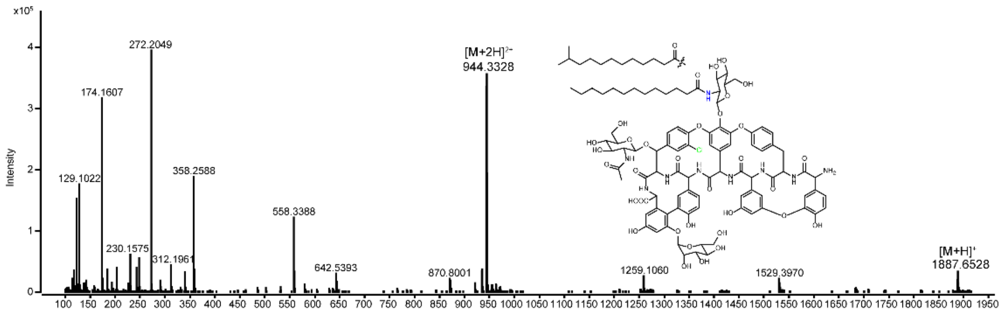
x10⁵
Intensity
272.2049
[M+2H]²⁺
944.3328
174.1607
129.1022
358.2588
558.3388
230.1575
312.1961
642.5393
870.8001
1259.1060
1529.3970
[M+H]⁺
1887.6528

j

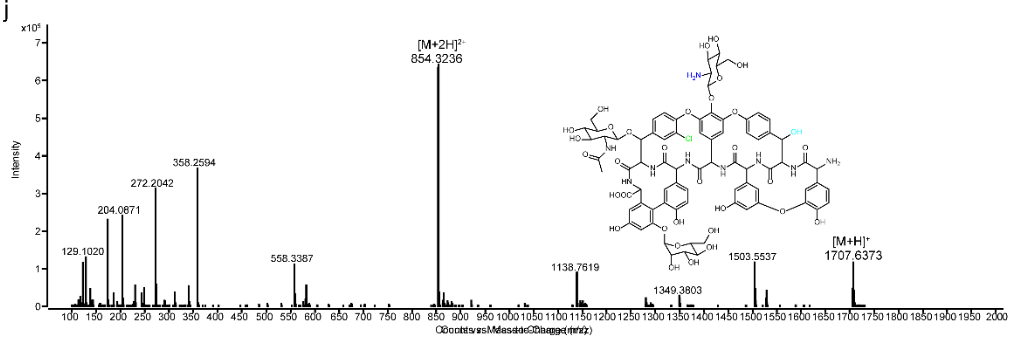
x10⁵
Intensity
[M+2H]²⁺
854.3236
358.2594
272.2042
204.0871
129.1020
558.3387
1138.7619
1349.3803
1503.5537
[M+H]⁺
1707.6373
Counts vs. Mass-to-Charge (m/z)

k

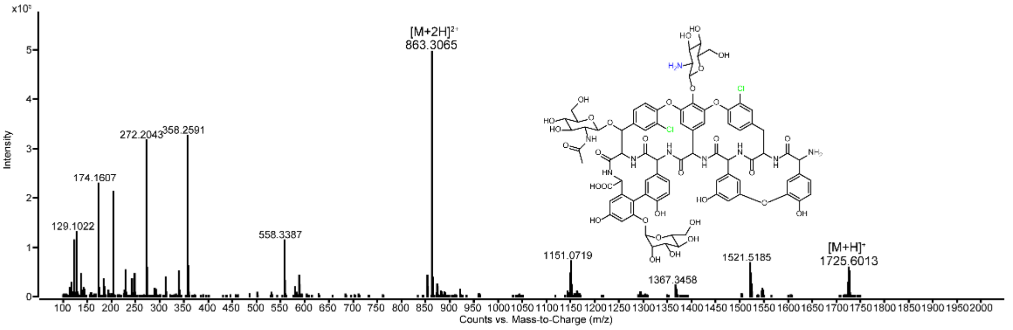
x10⁵
Intensity
[M+2H]²⁺
863.3065
272.2043
358.2591
174.1607
129.1022
558.3387
1151.0719
1367.3458
1521.5185
[M+H]⁺
1725.6013
Counts vs. Mass-to-Charge (m/z)

l

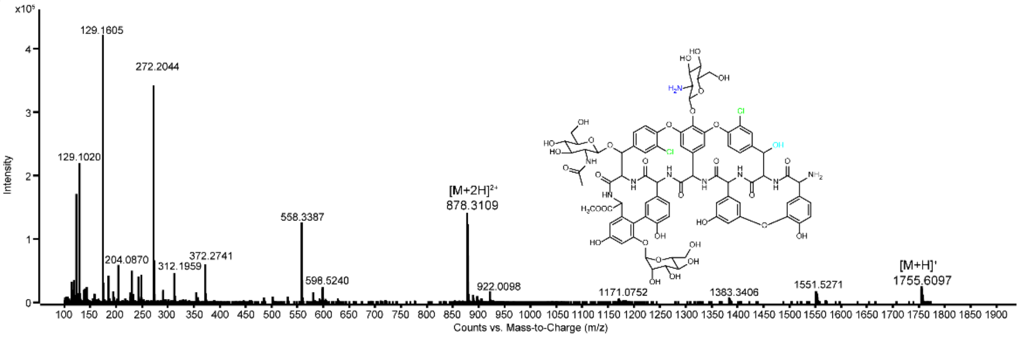
x10⁵
Intensity
129.1605
272.2044
129.1020
558.3387
[M+2H]²⁺
878.3109
204.0870
312.1959
372.2741
598.5240
922.0098
1171.0752
1383.3406
1551.5271
[M+H]⁺
1755.6097
Counts vs. Mass-to-Charge (m/z)

m

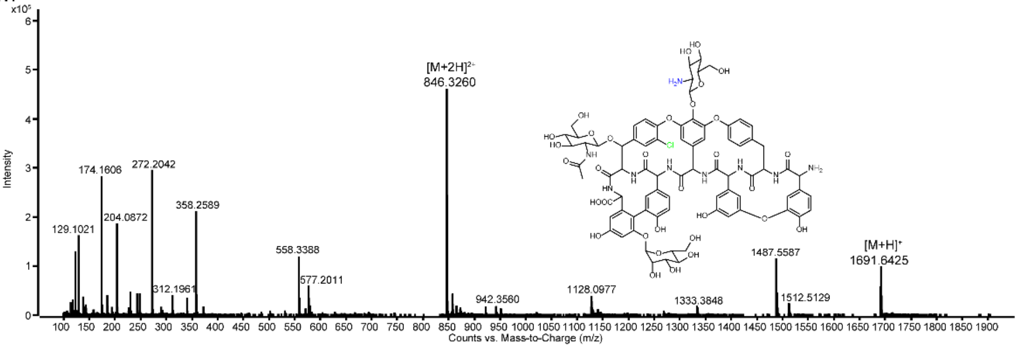
x10⁵
Intensity
[M+2H]²⁺
846.3260
174.1606
272.2042
358.2589
129.1021
204.0872
558.3388
577.2011
312.1961
942.3560
1128.0977
1333.3848
1487.5587
1512.5129
[M+H]⁺
1691.6425
Counts vs. Mass-to-Charge (m/z)

n

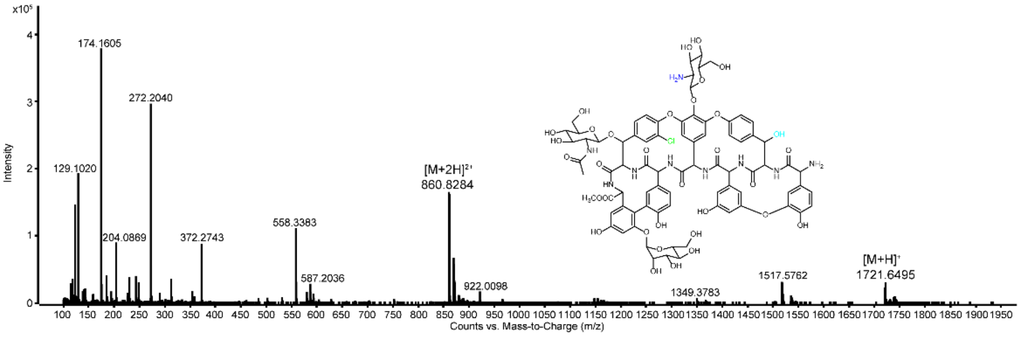
x10⁵
Intensity
174.1605
272.2040
129.1020
[M+2H]²⁺
860.8284
558.3383
204.0869
372.2743
587.2036
922.0098
1349.3783
1517.5762
[M+H]⁺
1721.6495
Counts vs. Mass-to-Charge (m/z)

o

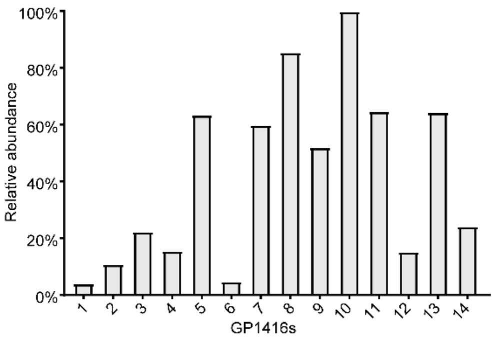
Relative abundance
GP1416s

Supplementary Fig. 17. Mass spectra of GP1416 analogs identified in *S. coelicolor* M1154/pAMX4/pGP1416. Proposed chemical structures of each analog (a to n correspond to peaks 1 to 14 in Fig. 3b) are also shown according to the characterized deacyl-GP1416 structure. The major structural difference between GP1416 and teicoplanin are: 1) chlorination of AA2 and AA6 residues, 2) β-hydroxylation of AA2 residue, and 3) identity of the acyl chain on glucosamine attached to Hpg4. Relative abundance of each analog was shown in o as normalized by the amount of 10 determined by mass spectrometry.

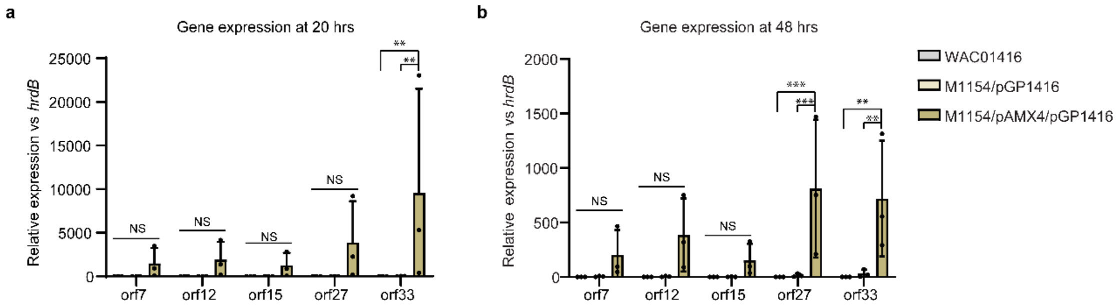

Supplementary Fig. 18. Transcriptional analysis of the GP1416 BGC. a-b, Relative expression of genes from GP1416 BGC sampled at 20 hrs (**a**) and 48 hrs (**b**). GP1416 BGC is transcriptionally inactive in both the wild type strain, *Amycolatopsis* sp. WAC01416, and the heterologous expression strain, *S. coelicolor* M1154/pGP1416. However, GP1416 BGC is actively transcribed in the GPAHex production chassis. Mean and standard deviation of biological triplicates (n=3) are shown with expression given relative to the housekeeping gene *hrdB*. Multiple comparison significance was tested to **P=0.0047 (**a**) and ***P=0.0009 or **P=0.0041 (**b**) by two-way ANOVA with Turkey's post hoc analysis. NS, not significant. Similar results (**a-b**) were obtained from two independent experiments.

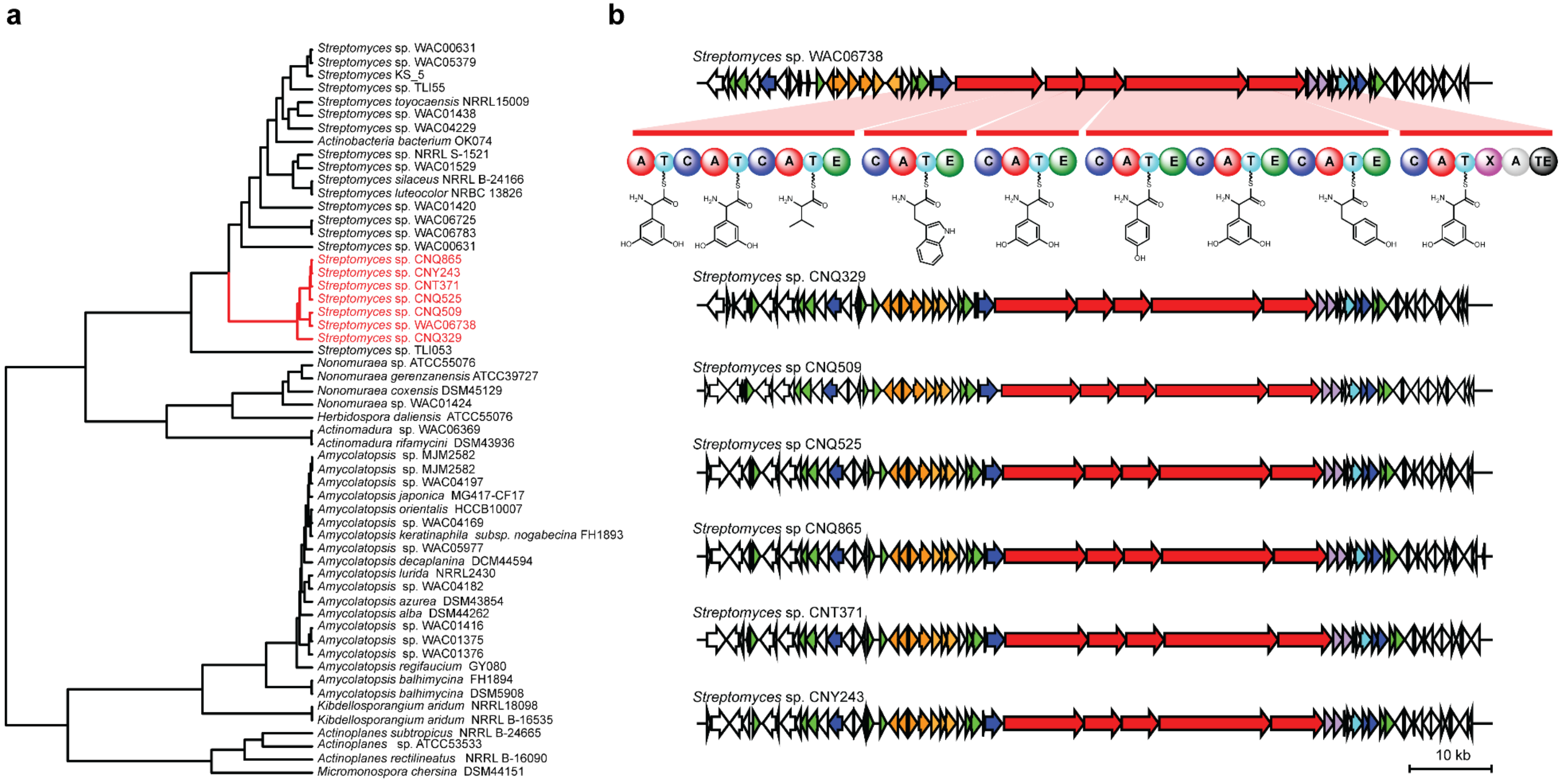

Supplementary Fig. 19. In silico analysis of GP6738 related BGCs. a, Concatenated TIGRFAM core-gene phylogeny of species with GPA BGCs from http://github.com/waglecn/GPA_evolution.git. Species with GP6738 and related BGCs form a monophyletic clade distinct from other GPA producing Streptomyces highlighted in red1. b, Genes organization of GP6738 related BGCs. Gene organization of the GP6738 BGC is almost identical in the six Streptomyces strains. Domain organization of the NRPS assembly line and predicted amino acid building blocks according to the preference of each A domain are also shown.

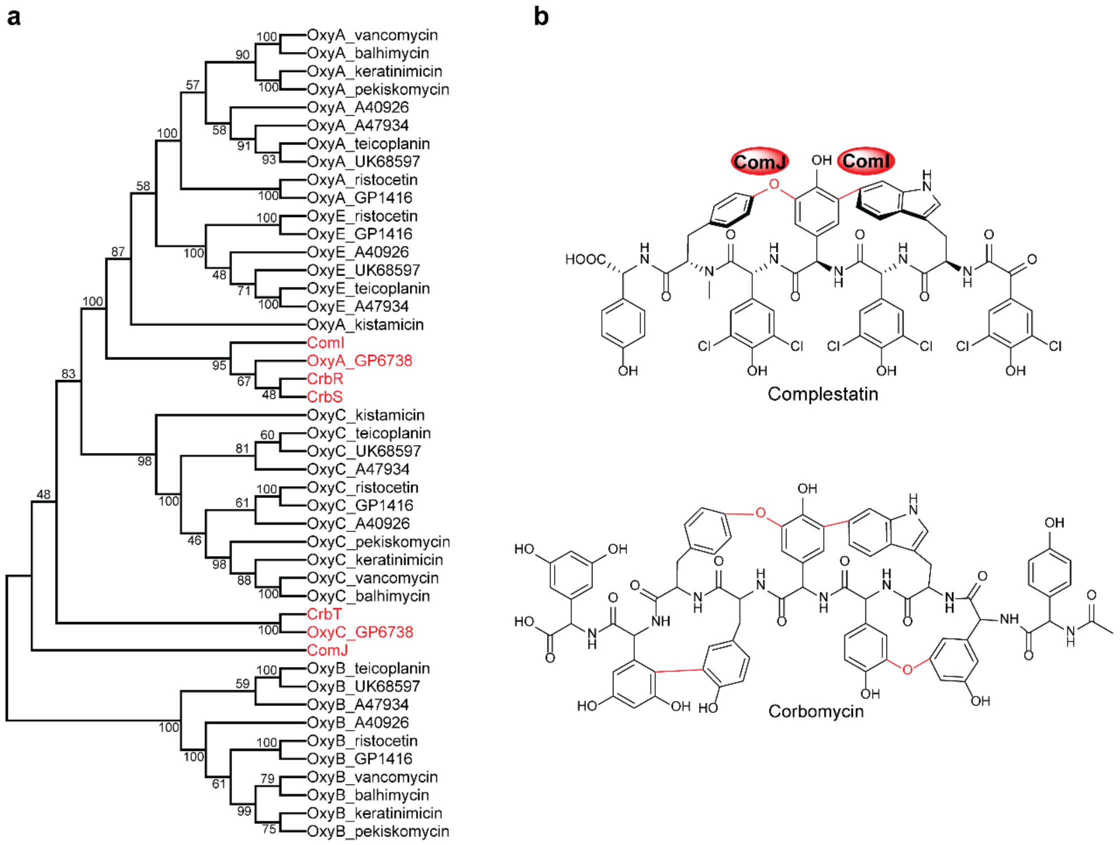

Supplementary Fig. 20. Phylogenetic analysis of P450 monooxygenases in GPA BGCs. a, The maximum likelihood bootstrap consensus tree of P450s in GPA BGCs arbitrarily rooted at OxyB. The two OxyA homologs from *crb* BGC cluster with OxyA from GP6738 BGC and ComI, forming a monophyletic clade distinct from OxyA and OxyE. The OxyC homolog from *crb* BGC clustered with the OxyC from GP6738 are closely related, but along with ComJ are distinct from the other P450s. **b**, Chemical structures of complestatin and corbomycin. ComJ and ComI catalyze the Tyr6-O-Hpg4 and Hpg4-Trp2 cross-links, respectively, during complestatin biosynthesis.

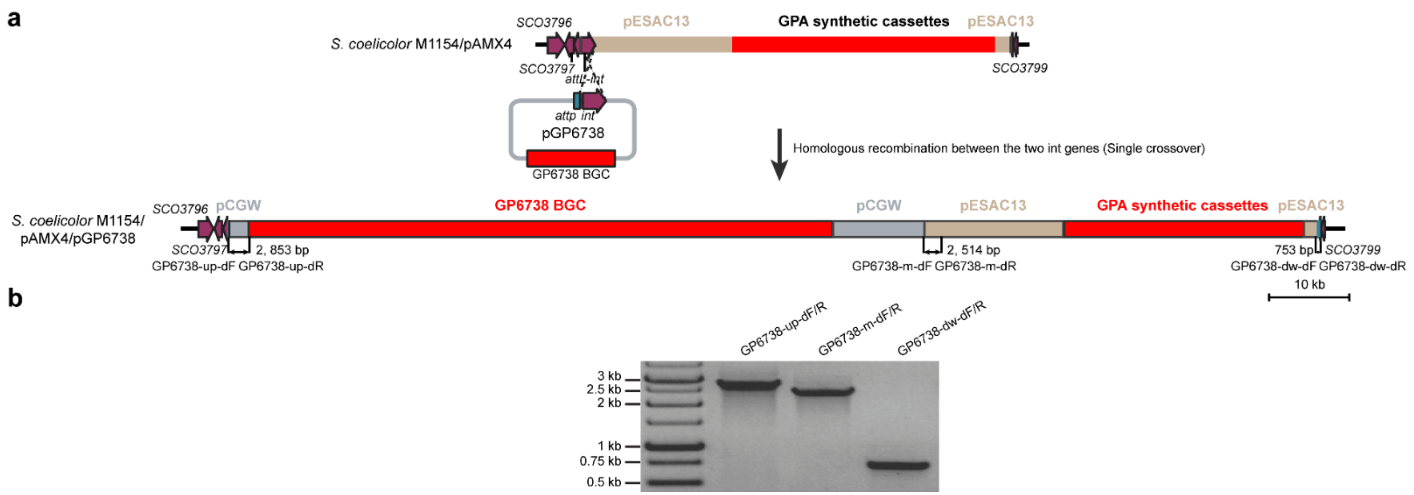

Supplementary Fig. 21. Integration of pGP6738 into the chromosome of *S. coelicolor* M1154/pAMX4. a, Schematic representation of pGP6738 insertion into the chromosome of *S. coelicolor* M1154/pAMX4 through homologous recombination between *attL-int*. Three pairs of diagnostic primers, GP6738-up-dF/R, GP6738-m-dF/R, and GP6738-dw-dF/R, are shown that confirmed the site of integration through PCR. Color-coding is identical to Supplementary Fig. 5. **b**, Gel image of the PCR products amplified from the genome of *S. coelicolor* M1154/pAMX4/pGP6738 using the diagnostic primers in **a**. Similar results (**b**) were obtained from three independent experiments. Source data underlying Supplementary Figure 21b are provided as a Source Data file.

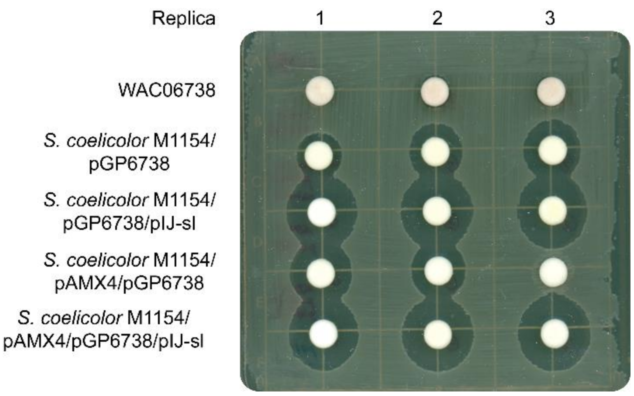

Supplementary Fig. 22. Bioactivity of GP6738 crude extracts. A) Bioassay of the crude extracts from the wild type strain *S.* sp. WAC06738, heterologous expression strains: *S. coelicolor* M1154/pGP6738 and *S. coelicolor* M1154/pAMX4/pGP6738 and the transcriptional regulators *strR* and *lmbU* over-expression heterologous strains: *S. coelicolor* M1154/pGP6738/pIJ-sl and *S. coelicolor* M1154/pAMX4/pGP6738/pIJ-sl. No zone of inhibition was observed for *S.* sp. WAC06738 crude extracts, while large inhibition halos were observed in both heterologous expression strains and transcriptional regulators over-expression heterologous strains. Source data are provided as a Source Data file.

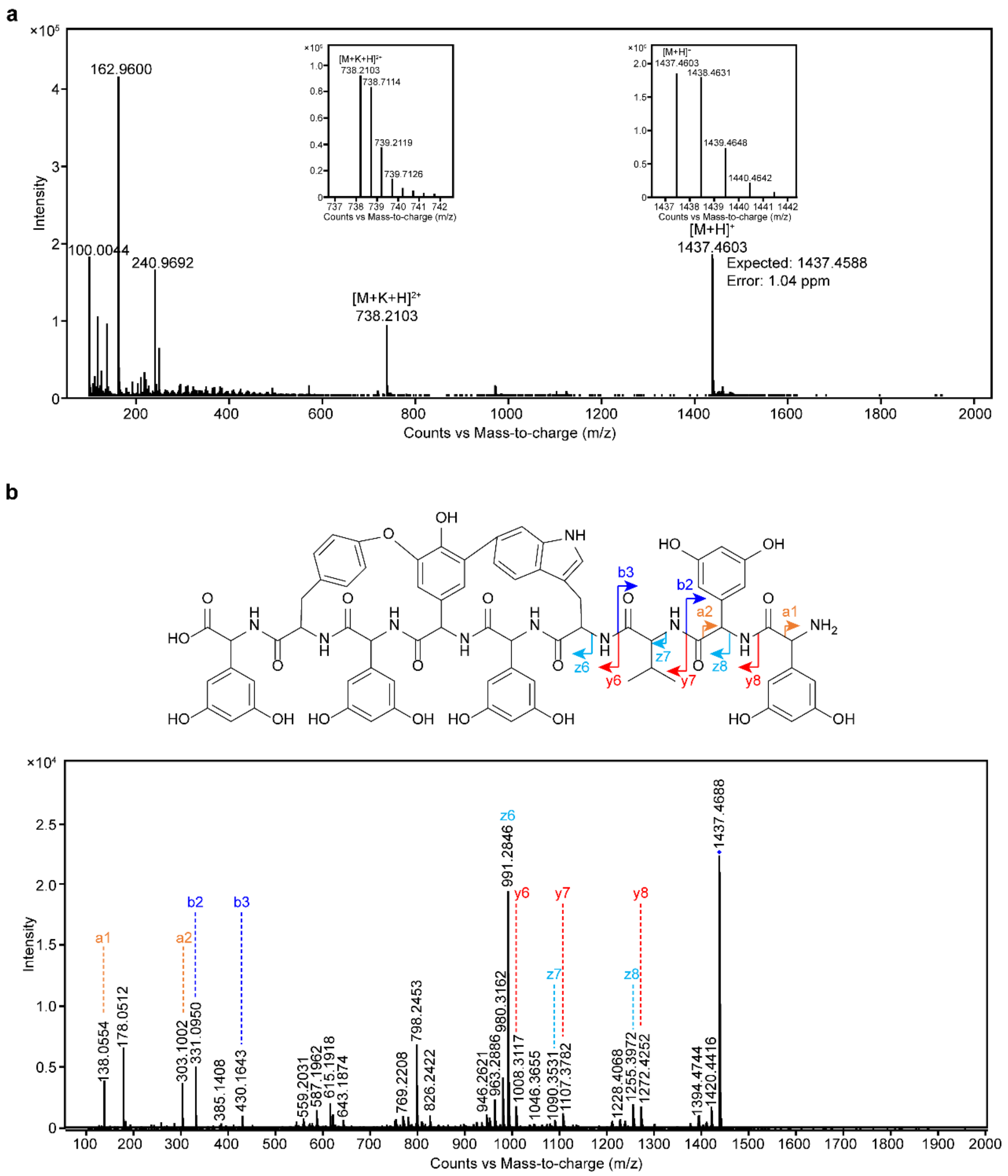

Supplementary Fig. 23. HR-MS and MS/MS of GP6738 determined by HRESI-Q-TOF mass spectrometry. a. HR-MS spectrum of GP6738. A singly charged ion of $[M+H]^{+}$=1437.4603 and a doubly charged ion of $[M+K+H]^{2+}$=738.2103 were detected (error 1.04 ppm). **b.** MS/MS production ions spectrum of GP6738. a, b, y, and z type production ions are colored orange, blue, red, and cyan, respectively.

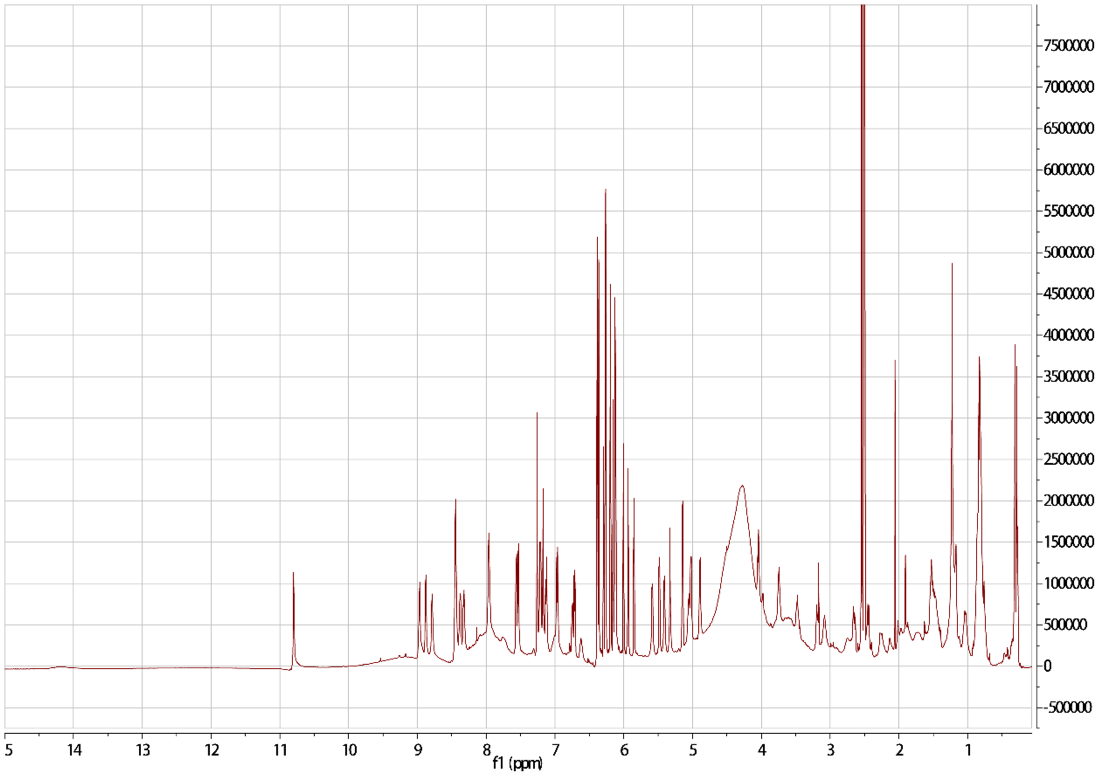

Supplementary Fig. 24. ^{1}H NMR spectrum of GP6738 in DMSO-*d6*.

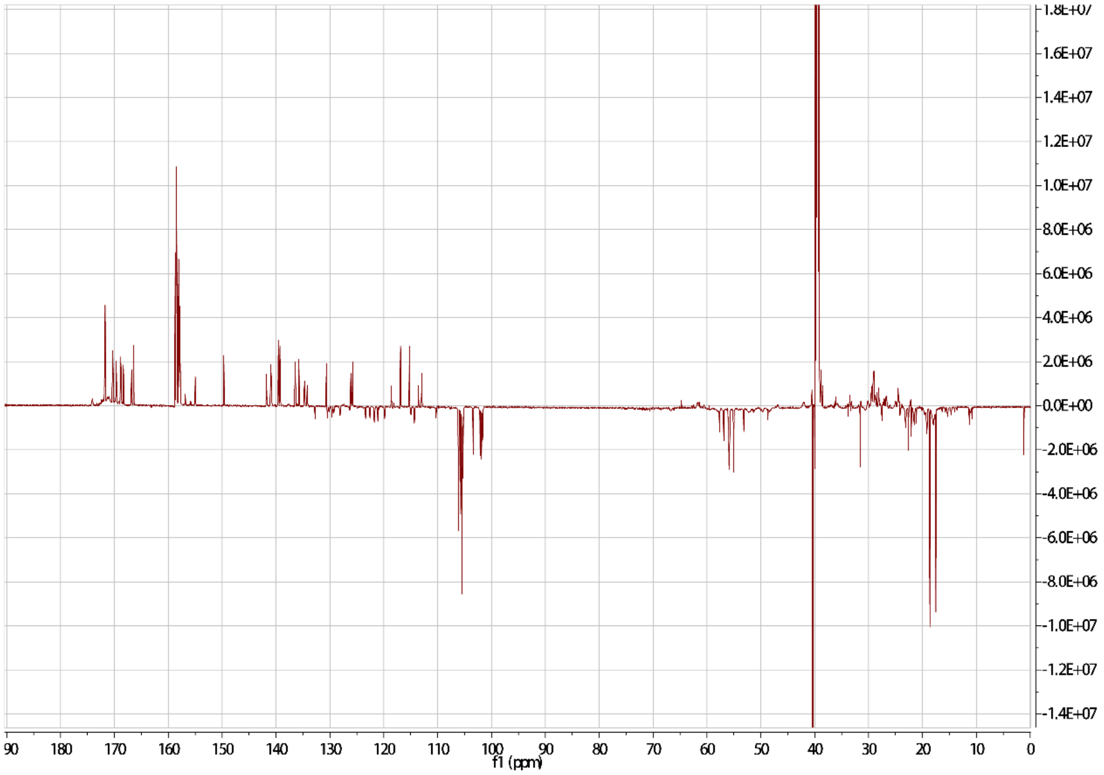

Supplementary Fig. 25. ^{13}C NMR spectrum of GP6738 in DMSO-*d6*.

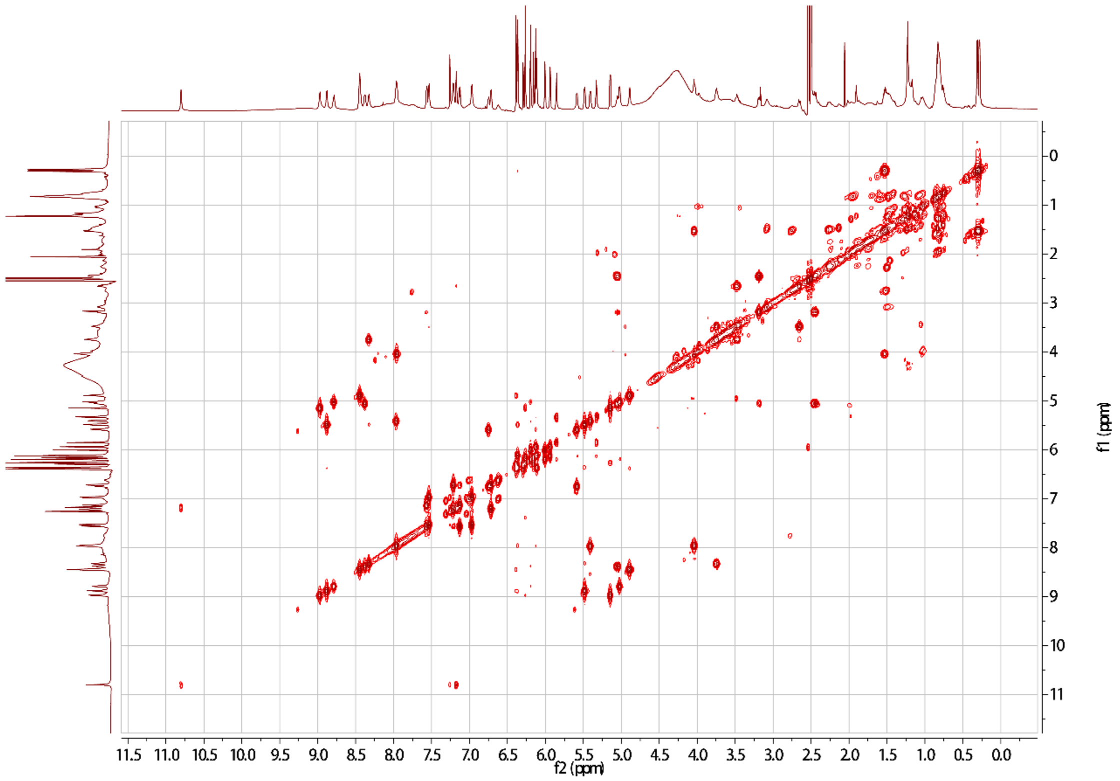

Supplementary Fig. 26. ^{1}H-^{1}H COSY NMR spectrum of GP6738 in DMSO-*d6*.

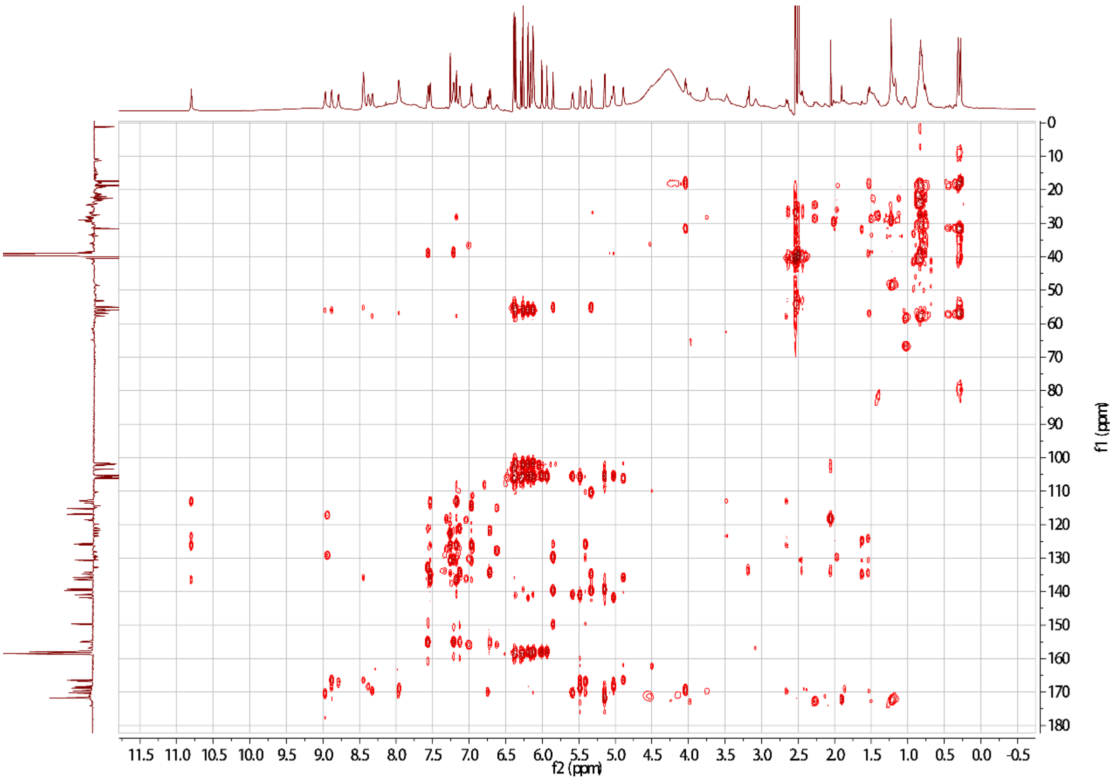

Supplementary Fig. 27. ^{1}H-^{13}C HMBC NMR spectrum of GP6738 in DMSO-*d6*.

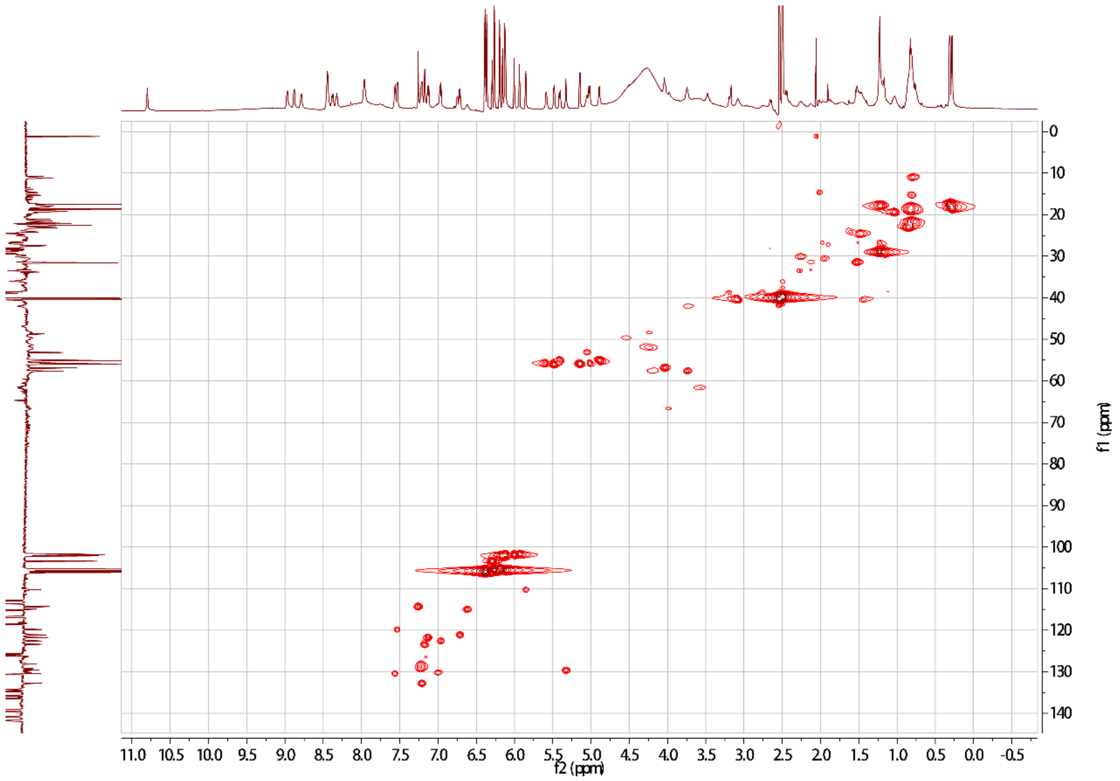

Supplementary Fig. 28. ^{1}H-^{13}C HSQC NMR spectrum of GP6738 in DMSO-*d6*.

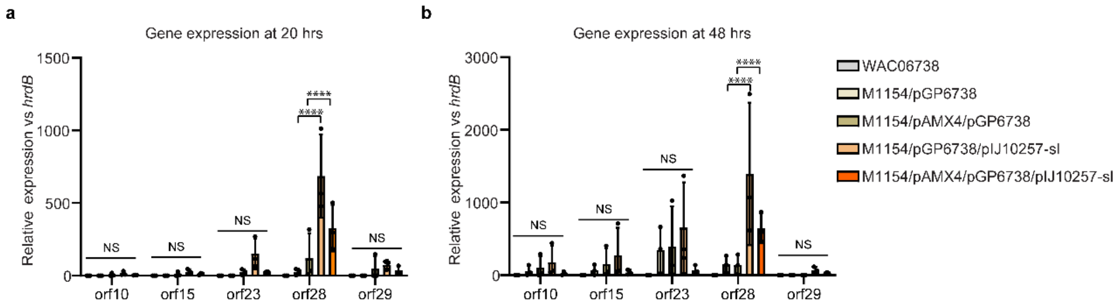

Supplementary Fig. 29. Transcriptional analysis of GP6738 BGC. **a-b**, Relative expression of genes from GP6738 BGC sampled at 20 hrs (**a**) and 48 hrs (**b**). GP6738 BGC is transcriptionally inactive in the wild type strain, *S.* sp. WAC06738. GP6738 BGC is actively transcribed in both *S. coelicolor* M1154 and the GPAHex production chassis *S. coelicolor* M1154/pAMX4. Over-expression of the pathway-situated regulators, *strR* and *lmbU*, further increased the transcriptional level of the genes from GP6738 BGC. Mean and standard deviation of biological triplicates (n=3) are shown, and expression is given in relation to the housekeeping gene *hrdB*. Multiple comparison significance was tested to ****P<0.0001 by two-way ANOVA with Turkey's post hoc analysis. NS, not significant. Similar results (**a-b**) were obtained from two independent experiments.

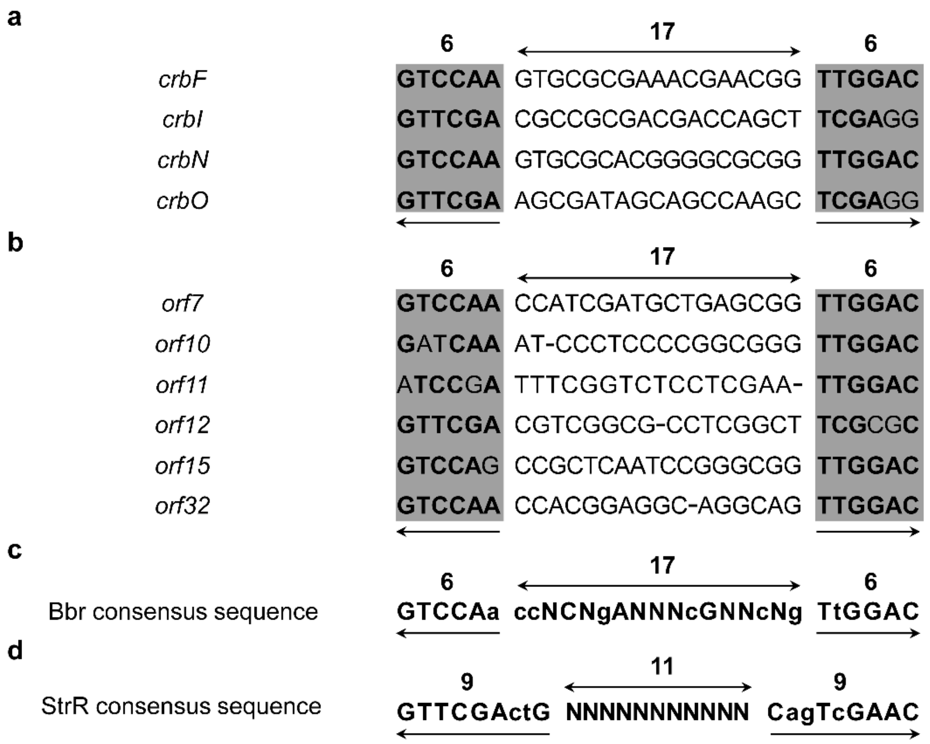

Supplementary Fig. 30. Putative StrR binding sites identified in *crb* and *GP1416* BGCs. a. Putative StrR binding sites identified in *crb* BGC. **b.** Putative StrR binding sites identified in GP1416 BGC. The genes harboring the putative StrR binding sites in their promoter region are shown on the left. Arrows indicate the inverted repeat sequences, double arrows indicate the spacing sequence. **c.** Consensus sequence of Bbr (StrR-like regulator from balhimycin BGC)[2]. **d.** Consensus sequence of StrR from the streptomycin BGC[3].

Supplementary Table 1. gblocks used for in this study.

Name	Sequence (5′-3′)	Description
crb-gblock	gcctcccatggtataaatagt**ggcgacggtgcccggttcggactctgctacctcgacctcgacggcttcaagaccgtcaa**GTTTAA**acacccaaaaccctcaaggccgttcgcacaagagtgcccctgaaaccgtt**AACtatgtcgaaagctacatataagga	*Hpa*I insertion site
gp1416-gblock	gcctcccatggtataaatagtggc**gtattgtcgccgatcaagtcagtccggtcgccaagccgaagatcagaatcctgtt**TAAA**ccgatcaaggtgcgcttcctcgtggtccggcaaggtccggacgtggctcggtt**AACtatgtcgaaagctacatataagga	*Hpa*I insertion site
gp6738-gblock	gcctcccatggtataaatagt**ggctacttcgagaccgagttcgagtccgagaaccgcaagtgggacgacgcgtacaag**TTTAA**acgcgaacgtcatccgcaagggcaaggccaccgagaaggaggagatcgcggcagc**Atatgtcgaaagctacatataagga	*Nde*I insertion site

Note: Capture sequences identical to that of target BGCs are shown in bold. Nucleotides underlined with wave lines indicates the presence of TIS site. Introduced restriction sites are labelled with double underscore.

Supplementary Table 2. Summary of the corbomycin biosynthetic gene cluster.

Gene	orf	Homolog	Proposed function
crbA	C 203-1084	LysR family transcriptional regulator	Transcriptional regulator
crbB	C 1168-3519	UvrA protein	Excinuclease ABC subunit A
crbC	C 3711-4865	Hmo	Phenylglycolate oxidase
crbD	C 4870-5988	HmaS	Hydroxyphenyl pyruvate dioxygenase
crbE	6162-7304	Pdh	Prephenate dehydrogenase
crbF	7457-9706	HpgT	Hpg/Dpg aminotransferase
crbG	9753-10901	DpgA	Dihydroxyphenylglycine synthase
crbH	10895-11614	DpgB	Enoyl-CoA hydratase
crbI	11611-12996	DpgC	Hydroxyacyl-dehydrogenase
crbJ	13007-13831	Abhydrolase	Hydrolase
crbK	13932-14156	StaE	MbtH
crbL	14198-16369	StaU	ABC transporter
crbM	16541-17854	VanS	Transmembrane histidine kinase
crbN	18023-29272	NRPS (Module 1-3)	Peptide synthase
crbO	29269-33885	NRPS (Module 4)	Peptide synthase
crbP	33954-48038	NRPS (Module 5-7)	Peptide synthase
crbQ	48121-59397	NRPS (Module 8-9)	Peptide synthase
crbR	59446-60651	OxyA	P450
crbS	60694-61806	OxyA	P450
crbT	61819-63024	OxyC	P450
crbU	63092-63310	Ferredoxin	Ferredoxin
crbV	63515-64114	-	Unknown
crbW	64111-65520	Peptidoglycan binding protein	Resistance
crbX	65517-66260	ABC transporter ATP binding protein	ABC transporter ATP binding protein
crbY	66257-67507	ABC transporter permease	ABC transporter permease
+1	C 67630-68628	Oxidoreductase	Oxidoreductase
+2	68731-69219	-	Unknown
+3	C 69263-69484	Dodecin	Unknown
+4	69735-71024	ABC transporter extraellular-binding protein	ABC transporter
+5	C 71035-71997	EgtD	Histidine methyltransferase
+6	C 71994-72749	EgtC	γ-glutamyl-hercynlcysteine sulfoxide hydrolase
+7	C 71749-74092	EgtB	Hercynine oxygenase
+8	C 74089-75477	EgtA	Glutamate-cysteine ligase

C: Complement strand.

Supplementary Table 3. Summary of the GP1416 biosynthetic gene cluster.

Gene	orf	Homolog	tei homologue	Proposed function
1	798-2039	-	-	Unknown
2	C 2425-2991	TetR family transcriptional regulator	Tei8	Transcriptional regulator
3	3110-3847	Abhydrolase	Tcp39	Hydrolase
4	3960-4997	VanH	Tcp2	D-lactate dehydrogenase
5	4999-6039	VanA	Tcp3	D-Ala-D-Lac ligase
6	6036-6644	VanX	Tcp4	D-Ala-D-Ala peptidase
7	7811-8608	StrR	Tcp28	StrR family transcriptional regulator
8	8788-9633	Pdh	-	Prephenate degydrogenase
9	9662-11726	StaU	Tcp16	ABC transporter
10	11719-17946	NRPS (module 1-2)	Tcp9	Peptide synthase
11	18018-21128	NRPS (module 3)	Tcp10	Peptide synthase
12	21638-33706	NRPS (module 4-6)	Tcp11	Peptide synthase
13	33963-39446	NRPS (module 7)	Tcp12	Peptide synthase
14	39458-39667	StaE	Tcp13/Tcp17	MbtH
15	39801-40976	OxyA	Tcp18	P450
16	41000-42157	OxyE	Tcp19	P450
17	42147-43343	OxyB	Tcp20	P450
18	43454-44695	OxyC	Tcp22	P450
19	44736-46211	Halogenase	Tcp21	Halogenase
20	46292-47395	GtfA	Tcp8	Glycosyltransferase
21	47499-48674	GtfB	Tcp23	Glycosyltransferase
22	48708-49670	Dbv8	Tcp24	Acyltransferase
23	49714-50592	Dbv21	Tcp14	Deacetylase
24	50676-52337	Dbv20	Tcp15	Mannosyltransferase
25	52478-52951	Acyl-CoA thioesterase	-	4-HB-CoA thioesterase
26	C 53272-54576	HpgT	Tcp36	Hpg/Dpg aminotransferase
27	54722-55555	Bhp	-	Hydrolase
28	55552-57285	BpsD	-	Peptide synthase
29	57300-58490	OxyD	-	P450
30	58647-59651	HmaS	Tcp37	Hydroxyphenyl pyruvate dioxygenase
31	59648-60724	Hmo	Tcp38	Phenylglycolate oxidase
32	60825-62714	StaN	Tcp34	Na^{+}-H^{+} antiporter
33	62328-63446	DpgA	Tcp30	Dihydroxyphenylglycine synthase
34	63440-64102	DpgB	Tcp31	Enoyl-CoA hydratase
35	64099-65313	DpgC	Tcp32	Hydroxyacyl-dehydrogenase
36	65310-66106	DpgD	Tcp33	Enoyl-CoA hydratase
37	66269-67345	DAHPS	Tcp27	DAHP synthase

C: Complement strand.

Supplementary Table 4. Summary of the GP6738 biosynthetic gene cluster.

Gene	orf	Homolog	Proposed function
1	625-1014	-	Unknown
2	C 1049-2218	VanS	Transmembrane histidine kinase
3	C 2199-2852	VanT	Two component response regulator
4	C 3030-4286	ABC transporter permease	Transporter
5	C 4283-5011	ABC transporter ATP binding protein	Transporter
6	C 5008-6390	Peptidoglycan binding protein	Resistance
7	C 6387-6929	-	Unknown
8	C 7084-7365	Ferrodoxin	Ferrodoxin
9	C 7390-8604	OxyC	P450
10	C 8613-9776	OxyA	P450
11	C 9907-16452	NRPS (Module 9)	Peptide synthase
12	C16466-30274	NRPS (Module 6-8)	Peptide synthase
13	C 30362-34945	NRPS (Module 5)	Peptide synthase
14	C 34942-39534	NRPS (Module 4)	Peptide synthase
15	C 39573-49562	NRPS (Module 1-3)	Peptide synthase
16	C 49792-51930	StaU	ABC transporter
17	C 51968-52198	StaE	MbtH
18	C 52348-53649	VanS	Transmembrane histidine kinase
19	C 53868-54344	VanT	Two component response regulator
20	C 54557-55360	Abhydrolase	Hydrolase
21	C 55470-56774	DpgC	Hydroxylacyl-dehydrogenase
22	C 56771-57454	DpgB	Enoyl-CoA hydratase
23	C 57448-58596	DpgA	Dihydroxyphenylglycine synthase
24	C 58708-60102	HpgT	Hpg/Dpg aminotransferase
25	C 60285-61406	Pdh	Prephenate dehydrogenase
26	61625-62731	HmaS	Hydroxyphenyl pyruvate dioxygenase
27	62712-63845	Hmo	Phenylglycolate oxidase
28	C 64114-64998	StrR transcriptional regulator	Transcriptional regulator
29	C 65746-66435	LmbU transcriptional regulator	Transcriptional regulator
30	66638-67105	-	Unknown
31	C 67130-68137	RNA polymerase Sig-70 factor	RNA polymerase
32	68270-69493	Oxidoreductase	Oxidoreductase
33	69632-71398	ABC transporter	ABC transporter
34	71428-72657	-	Unknown
35	72803-74053	LytS histidine kinase	Transmembrane histidine kinase
36	74101-74886	LytR response regulator	Two component response regulator
37	74888-75505	-	Unknown
38	75519-77312	Cation/acetate symporter	Cation/acetate symporter
39	77597-77971	-	Unknown

C: Complement strand

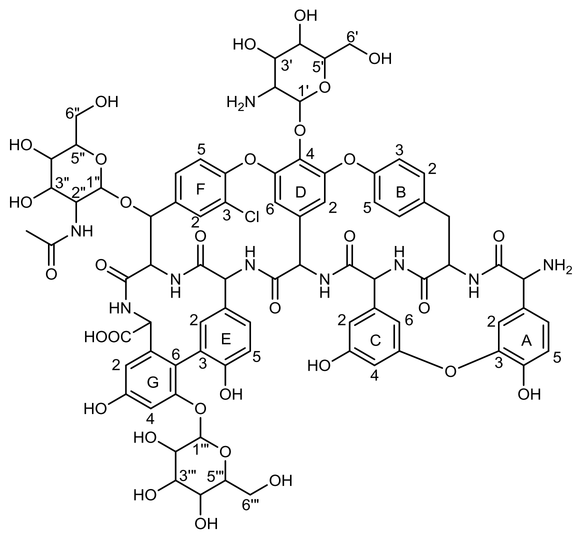

Supplementary Table 5. H and C NMR Data of deacyl-gp1416.

No.	δ_H	δ_C	No.	δ_H	δ_C
A-1		123.2	E-aCH	4.45 (brs)	54.1
A-2	6.75 (brs)	120.2	E-C=O		170.9
A-3		143.7	F-1		137.9
A-4		148.5	F-2	7.69 (brs)	128.6
A-5	6.97 (brd, *J* = 8.4 Hz)	118.7	F-3		127.5
A-6	7.07 (brd, *J* = 8.4 Hz)	127.7	F-4		149.3
A-αCH	5.19 (brs)	55.9	F-5	7.35 (brd, *J* = 8.1 Hz)	123.9
A-C=O		168.5	F-6	7.24 (brd, *J* = 8.1 Hz)	127.7
B-1		133.4	F-αCH	4.20 (brs)	62.2
B-2	7.15 (brd, *J* = 8.1 Hz)	130.5	F-βCH	5.47 (overlapped)	78.4
B-3	7.18 (brd, *J* = 8.1 Hz)	124.3	F-C=O		168.7
B-4		154	G-1		134.9
B-5	6.91 (brd, *J* = 8.4 Hz)	122.3	G-2	6.53 (brs)	108.3
B-6	7.49 (brd, *J* = 8.4 Hz)	132.1	G-3		156.8
B-αCH	5.08 (dd, *J* = 4.1, 4.4 Hz)	56.1	G-4	6.86 (brs)	102.8
	3.05 (brd, *J* = 14.2 Hz),				
B-βCH2	3.24 (brd, *J* = 14.2 Hz)	36.6	G-5		154
B-C=O		171.8	G-6		120.4
C-1		139.5	G-αCH	4.75 (overlap)	56.7
C-2	6.48 (brs)	109.9	G-C=O		173.8
C-3		159.7	1'	5.51 (overlapped)	100.2
C-4	6.63 (brs)	105.8	2'	3.41 (m, overlapped)	56.2
C-5		157.4	3'	3.81 (m, overlapped)	71.9
C-6	6.38 (brs)	105.4	4'	3.65 (m, overlapped)	69.1
C-aCH	5.45 (overlapped)	58.2	5'	3.46 (m, overlapped)	76.6
C-C=O		169.9	6'	3.80 (2H, m, overlapped)	59.9
D-1		133.7	1"	4.52 (d, *J* = 8.1 Hz)	100.4
D-2	5.84 (brs)	108.7	2"	3.69 (m, overlapped)	55.6
D-3		153.2	3"	3.44 (m, overlapped)	73.6
D-4		132	4"	3.41 (m, overlapped)	69.4

D-5		151.7	5''	3.29 (m, overlapped)	75.7
D-6				3.65 (m, overlapped),	
	5.11 (brs)	104	6''	3.84 (m, overlapped)	60.6
D-αCH	5.54 (brs)	54.9	*CH3*-C=O	1.94 (3H, s)	22.3
D-C=O		169.7	CH3-*C=O*		174.5
E-1		125.7	1'''	5.51 (overlapped)	96.6
E-2	7.00 (brs)	135.7	2'''	3.27 (m, overlapped)	69.9
E-3		154.5	3'''	3.59 (m, overlapped)	69.8
E-4		120.4	4'''	3.60 (m, overlapped)	66.3
E-5	6.87 (brd, J = 8.4 Hz)	117.3	5'''	3.60 (m, overlapped)	73
				3.65 (m, overlapped),	
E-6	6.93 (brd, J = 8.4 Hz)	127.2	6'''	3.84 (m, overlapped)	60.5

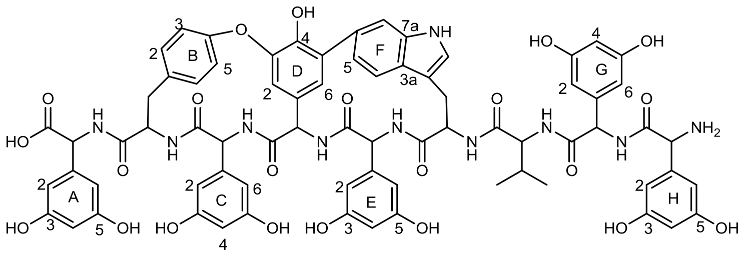

Supplementary Table 6. H and C NMR Data of GP6738.

No.	δ_H	δ_C	No.	δ_H	δ_C
A-1		139.28	E-C=O		170.32
A-2, 6	6.26 (2H, brd, *J* = 1.85 Hz)	105.46	E-NH	6.75 (d, *J* = 8.42 Hz)	
A-3, 5		158.51	F-1NH	10.80 (br s)	
A-4	6.16 (brt, *J* = 1.85 Hz)	102.12	F-2	7.17 (br s)	123.39
A-αCH	5.15 (d, *J* = 7.81 Hz)	55.92	F-3		112.93
A-C=O		171.73	F-3a		126.11
A-NH	8.97 (d, *J* = 7.81 Hz)		F-4	7.53 (d, *J*=7.49 Hz)	119.83
B-1		134.22	F-5	6.97 (d, *J*=7.49 Hz)	122.57
B-2	7.56 (d, *J* = 8.37 Hz)	130.44	F-6		134.72
B-3	7.13 (br d, *J* = 8.37 Hz)	121.77	F-7	7.26 (s)	114.31
B-4		155.00	F-7a		136.43
B-5	6.72 (br d, J = 8.19 Hz)	121.11	F-αCH	3.75 (br dd, *J*=10.22, 7.49 Hz)	57.66
				2.65 (brd, *J*=11.19 Hz),	
B-6	7.21 (br d, J = 8.19 Hz)	132.75	F-βCH2	3.48 (br dd, *J*=11.19, 10.22 Hz)	28.12
B-αCH	5.05 (overlapped)	53.17	F-C=O		169.71
	3.19 (brd, *J* = 10.35 Hz),				
B-βCH2	2.45 (m, overlapped)	38.81	F-NH	8.33 (brd, *J*=7.49 Hz)	
B-C=O		170.31	Val-αCH	4.04 (dd, *J*=7.85, 7.52 Hz)	56.88
C-1,		141.77	Val-βCH	1.53 (m)	31.56
C-2,6	6.19 (2H, brd, *J* = 1.71 Hz)	105.32	Val-Me1	0.31 (3H, d, *J*=6.52 Hz)	17.51
C-3,5		157.98	Val-Me2	0.29 (3H, d, *J*=6.52 Hz)	18.64
C-4,	6.01 (brt, *J* = 1.71 Hz)	101.83	Val-C=O		169.63
C-aCH	5.02 (d, *J* = 7.87 Hz)	55.73	Val-NH	7.96 (m, overlapped)	
C-C=O		168.34	G-1		140.95
C-NH	8.79 (d, *J* = 7.87 Hz)		G-2, 6	6.37 (2H, brd, *J* = 1.73 Hz)	105.72
D-1		125.79	G-3, 5		158.06
D-2	5.33 (br s)	129.61	G-4	6.12 (brt, *J* = 1.73 Hz)	101.86
D-3		149.70	G-αCH	5.48 (d, *J* = 8.06 Hz)	56.03
D-4		139.59	G-C=O		168.81
D-5		130.72	G-NH	8.88 (d, *J* = 8.06 Hz)	
D-6	5.85 (br s)	110.29	G-1		140.95
D-αCH	5.41(d, *J* = 8.67 Hz)	55.04	H-1		135.79
D-C=O		166.81	H-2, 6	6.39 (2H, brd, *J* = 1.85 Hz)	106.11
D-NH	7.97 (m, overlapped)		H-3, 5		158.71
E-1,		140.83	H-4	6.29 (brt, *J* = 1.85 Hz)	103.40
E-2,6	6.13 (2H, brd, *J* = 1.64 Hz)	105.51	H-αCH	4.89 (brd, *J* = 4.35 Hz)	55.07

E-3,5		157.89	H-C=O		166.44
E-4,	5.94 (brt, *J* = 1.64 Hz)	101.65	H-NH2	8.45 (2H, brd, *J* = 4.35 Hz)	
E-aCH	5.59 (d, *J* = 8.42 Hz)	55.84			

Supplementary Table 7. MICs of GP6738.

Strains	Compounds (μg/mL)			
	Complestatin	Corbomycin	GP6738	Vancomycin
E. coli BW25113	>256	>256	>256	256
E. coli BW25113Δ*bamB*Δ*tolC*	64	32	>256	8
S. aureus ATCC29213	2	1	4	1
MRSA USA300	2	1	4	1
Vancomycin intermediate MRSA Mu50	0.5	1	2-4	8
hVISA MRSA Mu3	0.5	1	4	2
B. subtilis 168	0.5	1	4	0.25
E. faecium ATCC19434	64	4	64	2
E. faecium ATCC70022 (VREA)	64	4	64	>256
E. faecium ATCC29212	32	4	64	2
E. faecium ATCC51299 (VREB)	32	4	64	32
S. venezuelae ATCC10712	2	2	16	0.25
S. coelicolor M1154	4	4	16	256
S. coelicolor M1154/pAMX4/pGP6738	ND	4	16	256

ND: Not determined.

Supplementary References

1. Waglechner N, McArthur AG, Wright GD. Phylogenetic reconciliation reveals the natural history of glycopeptide antibiotic biosynthesis and resistance. *Nat Microbiol* **4**, 1862-1871 (2019).
2. Shawky RM, *et al.* The border sequence of the balhimycin biosynthesis gene cluster from Amycolatopsis balhimycina contains bbr, encoding a StrR-like pathway-specific regulator. *Journal of Molecular Microbiology and Biotechnology* **13**, 76-88 (2007).
3. Retzlaff L, Distler J. The regulator of streptomycin gene expression, StrR, of Streptomyces griseus is a DNA binding activator protein with multiple recognition sites. *Molecular Microbiology* **18**, 151-162 (1995).
4. MacNeil DJ, Gewain KM, Ruby CL, Dezeny G, Gibbons PH, MacNeil T. Analysis of *Streptomyces avermitilis* genes required for avermectin biosynthesis utilizing a novel integration vector. *Gene* **111**, 61-68 (1992).
5. Gust B, Challis GL, Fowler K, Kieser T, Chater KF. PCR-targeted *Streptomyces* gene replacement identifies a protein domain needed for biosynthesis of the sesquiterpene soil odor geosmin. *Proceedings of the National Academy of Sciences of the United States of America* **100**, 1541-1546 (2003).
6. Tang X, *et al.* Identification of thiotetronic acid antibiotic biosynthetic pathways by target-directed genome mining. *ACS Chemical Biology* **10**, 2841-2849 (2015).
7. Gomez-Escribano JP, Bibb MJ. Engineering *Streptomyces coelicolor* for heterologous expression of secondary metabolite gene clusters. *Microbial Biotechnology* **4**, 207-215 (2011).
8. Yim G, Wang W, Thaker MN, Tan S, Wright GD. How to make a glycopeptide: A synthetic biology approach to expand antibiotic chemical diversity. *ACS Infect Dis* **2**, 642-650 (2016).
9. Jones AC, Gust B, Kulik A, Heide L, Buttner MJ, Bibb MJ. Phage p1-derived artificial chromosomes facilitate heterologous expression of the FK506 gene cluster. *PLoS ONE* **8**, e69319 (2013).
10. Hong HJ, Hutchings MI, Hill LM, Buttner MJ. The role of the novel Fem protein VanK in vancomycin resistance in *Streptomyces coelicolor*. *The Journal of Biological Chemistry* **280**, 13055-13061 (2005).
11. Datsenko KA, Wanner BL. One-step inactivation of chromosomal genes in *Escherichia coli* K-12 using PCR products. *Proceedings of the National Academy of Sciences of the United States of America* **97**, 6640-6645 (2000).
