# Supplementary material for: GPAHex-A synthetic biology platform for Type IV–V glycopeptide antibiotic production and discovery
Source: Nat Commun. 2020 Oct 16;11:5232. doi: 10.1038/s41467-020-19138-5 (PMC7567792; doi:10.1038/s41467-020-19138-5)
Supplement: Supplementary file 2 — Reporting Summary [file 41467_2020_19138_MOESM2_ESM.pdf]

nature research

Corresponding author(s): Gerard Wright

Last updated by author(s): Sep 17, 2020

Reporting Summary

Nature Research wishes to improve the reproducibility of the work that we publish. This form provides structure for consistency and transparency in reporting. For further information on Nature Research policies, see our Editorial Policies and the Editorial Policy Checklist.

Statistics

For all statistical analyses, confirm that the following items are present in the figure legend, table legend, main text, or Methods section.

n/a	Confirmed	
☐	☒	The exact sample size (*n*) for each experimental group/condition, given as a discrete number and unit of measurement
☐	☒	A statement on whether measurements were taken from distinct samples or whether the same sample was measured repeatedly
☐	☒	The statistical test(s) used AND whether they are one- or two-sided<br>*Only common tests should be described solely by name; describe more complex techniques in the Methods section.*
☒	☐	A description of all covariates tested
☒	☐	A description of any assumptions or corrections, such as tests of normality and adjustment for multiple comparisons
☐	☒	A full description of the statistical parameters including central tendency (e.g. means) or other basic estimates (e.g. regression coefficient) AND variation (e.g. standard deviation) or associated estimates of uncertainty (e.g. confidence intervals)
☐	☒	For null hypothesis testing, the test statistic (e.g. *F*, *t*, *r*) with confidence intervals, effect sizes, degrees of freedom and *P* value noted<br>*Give P values as exact values whenever suitable.*
☒	☐	For Bayesian analysis, information on the choice of priors and Markov chain Monte Carlo settings
☒	☐	For hierarchical and complex designs, identification of the appropriate level for tests and full reporting of outcomes
☒	☐	Estimates of effect sizes (e.g. Cohen's *d*, Pearson's *r*), indicating how they were calculated

Our web collection on statistics for biologists contains articles on many of the points above.

Software and code

Policy information about availability of computer code

Data collection	No software was used for data collection.
Data analysis	GraphPad Prism 7.0 for statistical analysis as mentioned in figure legends.<br>MEGA X for alignment and making tree of P450 proteins.<br>AntiSMASH 5.0 for analyzing GPA biosynthetic gene clusters.

For manuscripts utilizing custom algorithms or software that are central to the research but not yet described in published literature, software must be made available to editors and reviewers. We strongly encourage code deposition in a community repository (e.g. GitHub). See the Nature Research guidelines for submitting code & software for further information.

Data

Policy information about availability of data

All manuscripts must include a data availability statement. This statement should provide the following information, where applicable:

- Accession codes, unique identifiers, or web links for publicly available datasets
- A list of figures that have associated raw data
- A description of any restrictions on data availability

Genome sequences of Streptomyces sp. WAC01529, Streptomyces sp. WAC06738, Amycolatopsis sp. WAC01416, S. coelicolor M1154/pAMX4, and S. coelicolor M1154/pAMX4/pGP1416 are available in GenBank with accession numbers “NZ_CP029617.1 [https://www.ncbi.nlm.nih.gov/assembly/GCF_003945545.1/]”, “NZ_CP029618.1 [https://www.ncbi.nlm.nih.gov/assembly/GCF_003945505.1/]”, “NZ_QHHX00000000.1 [https://www.ncbi.nlm.nih.gov/nuccore/NZ_QHHX00000000.1]”, “JAATOK000000000.1 [https://www.ncbi.nlm.nih.gov/nuccore/JAATOK000000000.1]” and “CP050522.1 [https://www.ncbi.nlm.nih.gov/nuccore/CP050522.1]”. Concatenated TIGRFAM core-gene phylogeny of species with GPA BGCs shown in Supplementary Fig. 19 is available at http://github.com/waglecn/GPA_evolution.git. Data supporting the findings of this work are available within the paper and its Supplementary Information files. A reporting summary

for this article is available as a Supplementary Information file. The datasets and materials generated and analyzed during the current study are available from the corresponding author upon request. The source data underlying Figures 5a, as well as Supplementary Figures 2b, 3a-b, 4, 5b, 6, 8b, 21b, and 22 are provided as a Source Data file. Source data are provided with this paper.

Field-specific reporting

Please select the one below that is the best fit for your research. If you are not sure, read the appropriate sections before making your selection.

- [x] Life sciences
- [ ] Behavioural & social sciences
- [ ] Ecological, evolutionary & environmental sciences

For a reference copy of the document with all sections, see nature.com/documents/nr-reporting-summary-flat.pdf

Life sciences study design

All studies must disclose on these points even when the disclosure is negative.

Sample size	All the quantitation of glycopeptide antibiotics production in actinomycetes, bioassay and RT-PCR experiment for checking genes expression were performed in biological triplicate (n=3). Good replication can be seen from the biological triplicates and clear distinctions could be made with this sample size.
Data exclusions	No data point was excluded from analysis.
Replication	Enhanced production and activation of glycopeptide antibiotics were validated through bioactivity test against B. subtilis 168, metabolic analysis by HPLC or LC-MS, and gene expression analysis by RT-PCR. Similar results were produced from at least two independent experiments.
Randomization	No randomization was required as no experiments performed involved the allocation of samples to test groups.
Blinding	No blinding was required as no experiments performed involved the allocation of samples to test groups.

Reporting for specific materials, systems and methods

We require information from authors about some types of materials, experimental systems and methods used in many studies. Here, indicate whether each material, system or method listed is relevant to your study. If you are not sure if a list item applies to your research, read the appropriate section before selecting a response.

Materials & experimental systems

n/a	Involved in the study
x	Antibodies
x	Eukaryotic cell lines
x	Palaeontology and archaeology
x	Animals and other organisms
x	Human research participants
x	Clinical data
x	Dual use research of concern

Methods

n/a	Involved in the study
x	ChIP-seq
x	Flow cytometry
x	MRI-based neuroimaging
