# Supplementary material for: GPAHex-A synthetic biology platform for Type IV–V glycopeptide antibiotic production and discovery
Source: Nat Commun. 2020 Oct 16;11:5232. doi: 10.1038/s41467-020-19138-5 (PMC7567792; doi:10.1038/s41467-020-19138-5)
Supplement: Supplementary file 3 — Description of Additional Supplementary Files [file 41467_2020_19138_MOESM3_ESM.pdf]

Description of Additional Supplementary Files

Supplementary Data 1
Strains and plasmids used in this study.

Supplementary Data 2
Primers used in this study.
